# Deciphering the dual nature of nesfatin-1: a tale of zinc ion’s Janus-faced influence

**DOI:** 10.1186/s12964-024-01675-x

**Published:** 2024-05-29

**Authors:** Rafał Lenda, Lilia Zhukova, Andrzej Ożyhar, Dominika Bystranowska

**Affiliations:** 1 Department of Biochemistry, Molecular Biology and Biotechnology, Wrocław University of Science and Technology, Wybrzeże Wyspiańskiego 27, Wrocław, 50-370 Poland; 2 Institute of Biochemistry and Biophysics, Polish Academy of Sciences, Pawińskiego 5a, Warsaw, 02-106 Poland

**Keywords:** *Gallus gallus*, Chicken, Metal cation binding protein, Nesfatins, Metalloprotein, Neuropeptide, Satiety molecule, Hormone, Intrinsically disordered protein

## Abstract

**Background:**

Nucleobindin-2 (Nucb2) and nesfatin-1 (N1) are widely distributed hormones that regulate numerous physiological processes, from energy homeostasis to carcinogenesis. However, the role of nesfatin-2 (N2), the second product of Nucb2 proteolytic processing, remains elusive. To elucidate the relationship between the structure and function of nesfatins, we investigated the properties of chicken and human homologs of N1, as well as a fragment of Nucb2 consisting of N1 and N2 conjoined in a head-to-tail manner (N1/2).

**Results:**

Our findings indicate that Zn(II) sensing, in the case of N1, is conserved between chicken and human species. However, the data presented here reveal significant differences in the molecular features of the analyzed peptides, particularly in the presence of Zn(II). We demonstrated that Zn(II) has a Janus effect on the M30 region (a crucial anorexigenic core) of N1 and N1/2. In N1 homologs, Zn(II) binding results in the concealment of the M30 region driven by a disorder-to-order transition and adoption of the amyloid fold. In contrast, in N1/2 molecules, Zn(II) binding causes the exposure of the M30 region and its destabilization, resulting in strong exposure of the region recognized by prohormone convertases within the N1/2 molecule.

**Conclusions:**

In conclusion, we found that Zn(II) binding is conserved between chicken and human N1. However, despite the high homology of chicken and human N1, their interaction modes with Zn(II) appear to differ. Furthermore, Zn(II) binding might be essential for regulating the function of nesfatins by spatiotemporally hindering the N1 anorexigenic M30 core and concomitantly facilitating N1 release from Nucb2.

**Supplementary Information:**

The online version contains supplementary material available at 10.1186/s12964-024-01675-x.

## Background

**Fig. 1:**
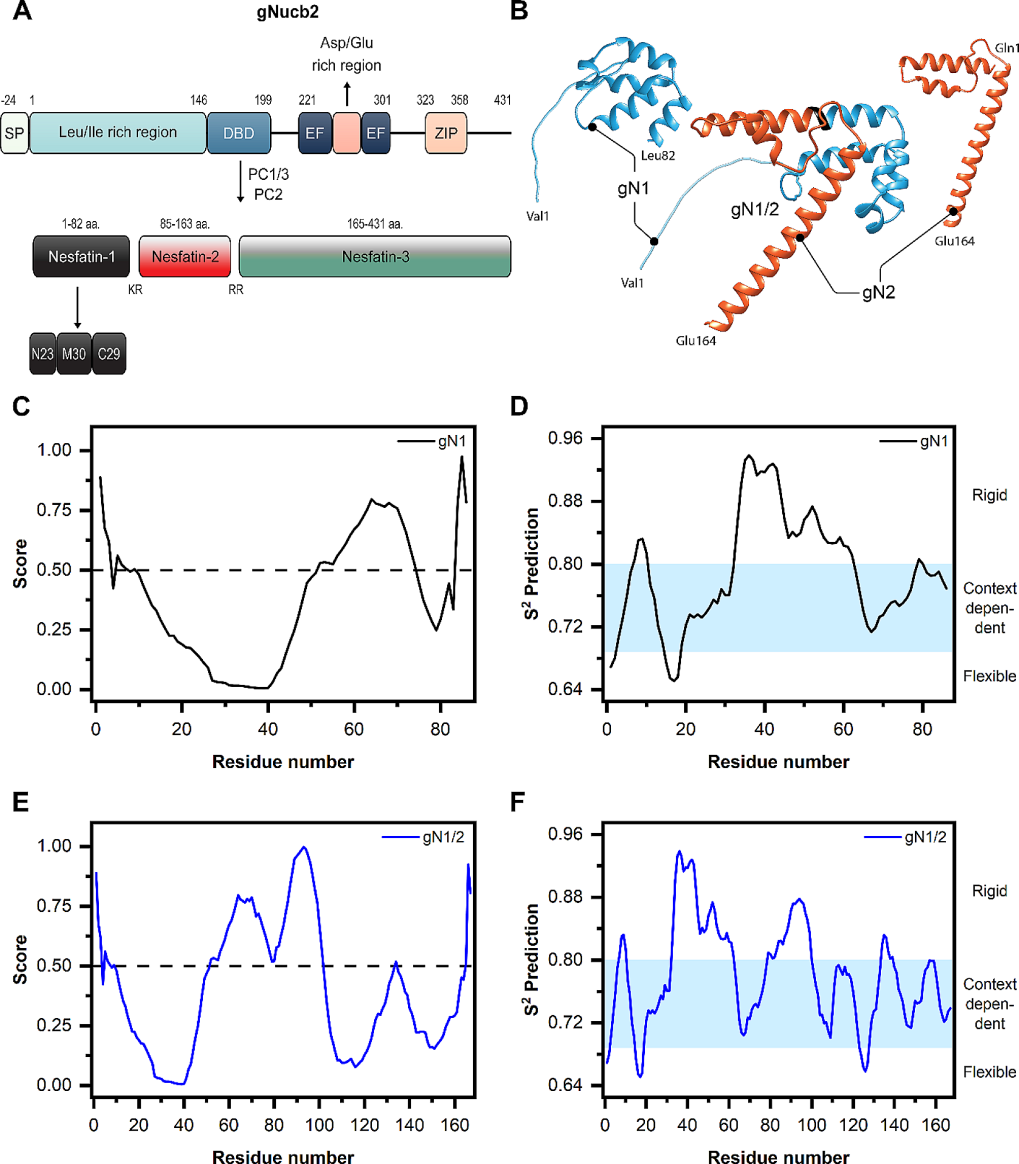
Proteolytic processing of chicken Nucb2 (gNucb2) to nesfatins and their disorder propensity analysis. (**A**) Representation of the multi-domain structure of gNucb2 and the products of PCs acting on the protein. (**B**) AlphaFold2 models of gN1, gN2, and gN1/2. Prediction of IDRs in (**C**) gN1 and (**E**) gN1/2 with the PONDR algorithm. Scores above the 0.5 threshold (dotted line) indicate disordered residues. Prediction of the backbone dynamics of (**D**) gN1 and (**F**) gN1/2 with the DynaMine algorithm

Nesfatin-1 (N1) is a leptin-independent peptide hormone displaying strong anorexigenic properties after central and peripheral administration [1]. In vivo, N1 is released from the precursor protein Nucleobindin-2 (Nucb2) by specific prohormone convertases (PCs) [2]. During the proteolytic processing of Nucb2 by PCs, two additional products are formed (Fig. 1A): nesfatin-2 (N2) and nesfatin-3 (N3) [2]. Among these three products, only N1 has been shown to exert a physiological function. The functions of the other peptides remain poorly understood [3]. Nucb2 structure is multi-domain, and consists of (Fig. 1A): short signal peptide (SP), Leu/Ile rich region, DNA binding domain (DBD), two EF-hand domains, region rich in the acidic amino acids, and leucine zipper motif (ZIP) [4]. The amino acid (aa) sequence of Nucb2 is highly conserved [5]. Another member of the highly conserved Nucleobindin family is Nucleobindin-1 (Nucb1), a Nucb2 paralog. The proteolytic processing of Nucb1 by PCs results in the formation of nesfatin-like peptide (NLP) [5]. In turn, the structure of N1/NLP is tripartite and comprises of the following fragments: an N-terminal (N23) fragment, a middle fragment (M30), and a C-terminal (C29) fragment. Shimizu et al. [6] demonstrated that the presence of the middle fragment, which shares sequence similarity with Agouti-related peptide (AgRP), is responsible for the strong inhibitory effect of food intake. Moreover, the aa sequence of the middle fragment of N1/NLP is also highly conserved between the paralogs [5]. Nucb2 and/or N1 (Nucb2/N1) are ubiquitous, and are found in both the central nervous system and peripheral tissues. In the brain, Nucb2/N1 have been found in the hypothalamus, nucleus of the solitary tract, amygdala, and more [7]. Other organs and tissues characterized by Nucb2/N1 expression include adipose tissue [8], heart [9], stomach [10], B cells of the pancreas [11], and testis [12]. The multi-domain structure in concert with the universal expression of Nucb2/N1 throughout the body underlines their high biological importance and supports their involvement in processes that extend far beyond the regulation of energy homeostasis such as the regulation of responses to stress and anxiety [13], epilepsy [14], depression [15], insulin release [16], and blood pressure regulation [17], to name a few. On the other hand, Nucb1 was shown to be involved in the inhibition of amyloid formation, which raises questions about the role of Nucb2 in these processes, given its high homology [18]. The WHO reported that more than 55 million people are diagnosed with dementia worldwide and that by 2050, this number will increase to 139 million [19]. For this reason, studies on the involvement of Nucb2/nesfatins and Nucb1/NLPs in neurodegenerative processes are vital. Furthermore, Nucb2/N1 exhibit tissue-specific and dual modes of action in carcinogenesis [20]. However, the understanding of the relationship between the structure and function of Nucb2/nesfatins is limited. Additionally, there has been a recent increase in research exploring the physiological effects of Nucb2/nesfatins and Nucb1/NLP in nonmammalian organisms, including goldfish [21], zebrafish [22], and frogs [23]. Tackling the structural and functional differences between the homologs and paralogs might provide more insight into the function of Nucleobindins and thus is of special importance. Additionally, the anorexigenic effects of Nucb2/nesfatins in birds are particularly interesting since adipose tissue storage is largely leptin independent, and there are differences in the regulation of energy metabolism between birds and mammals [24]. Moreover, Nucb2 was shown to be involved in the egg biomineralization process in chickens [25]. The conservation of the aa sequence between bird and mammalian nesfatins is exceptionally high. Nonetheless, there might be differences in the repertoire of functions among the species. In this context, we have shown that although human and chicken Nucb2 (hNucb2 and gNucb2, respectively) are intrinsically disordered proteins (IDPs) and display a mosaic structure with intertwined ordered and intrinsically disordered regions (IDRs), both proteins exist in solution in different conformations [26]. Moreover, both hNucb2 and gNucb2 were shown to bind Zn(II) and Ca(II) at the N- and C-termini, respectively [26, 27], with different affinities. Finally, we have provided insight into the structure of intrinsically disordered hN1, human N2 (hN2), and the two fragments conjoined in a head-to-tail manner (hN1/2), proven the conservation of Zn(II) sensing in the N-terminal fragments released from the precursor protein, and suggested a structural role for N2 [28].

In this paper, we focused on describing the detailed molecular characteristics of chicken and human homologs of N1 and N1/2 and investigated the structural effects of their interactions with Zn(II). Our findings demonstrate the persistence of the disordered character in both chicken and human N1, as well as the mosaic structure of both N1/2. We concluded that Zn(II) sensing is conserved between chicken and human homologs and might be a universal feature of nesfatins. Surprisingly, despite the very high homology of the aa sequences (based on the BLAST algorithm, identity ranging 85–87%), we observed startling differences between the homologs. The interactions of gN1 and gN1/2 with Zn(II) differed from those of the human homologs in terms of decreased cooperativity and increased propensity toward oligomerization and/or aggregation induced by the ion, thus indicating species-specific functions. Zn(II) also greatly destabilized both chicken and human N1 and N1/2 homologs, with gN1/2 being more sensitive to Zn(II). Surprisingly, the influence of Zn(II) on the M30 region was observed to be Janus-faced: upon Zn(II) binding, we observed structurization and concealment of the M30 anorexigenic core in gN1 and hN1, as well as exposition of the M30 region and the region recognized by the PCs in gN1/2 and hN1/2. This behavior clearly redefines the properties of N1 when covalently bound to N2. Hence, Zn(II) binding might be critical for the regulation of the function of nesfatins by spatiotemporally hindering the N1 anorexigenic effect and concomitantly facilitating its release from Nucb2 in Zn(II)-abundant milieus. Finally, chicken and human holo-N1 homologs were shown here to bind ThT in a Zn(II) concentration-dependent manner, implying the presence of an amyloid fold that could bind to and/or form amyloid fibrils. Hence, there is increasing evidence of the engagement of N1 in neurodegenerative processes.

## Materials and methods

### Chemicals

#### The buffers and solutions used were as follows

Buffer A1 (50 mM NaH_2_PO_4_ × 2H_2_O, pH 7.0; 300 mM NaCl), Buffer A2 (50 mM NaH_2_PO_4_ × 2H_2_O; 300 mM NaCl; 200 mM imidazole, pH 7.0), Buffer B1 (20 mM Tris-HCl, pH 7.5; 150 mM NaCl), Buffer B2 (20 mM Tris-HCl, pH 7.0; 150 mM NaCl), and Solution S (2 M glycine). All buffers were prepared at ambient temperature and filtered through a 0.22 μm filter.

#### Reagents

DNase I, RNase I, imidazole, EDTA, chloramphenicol, phenylmethylsulfonyl fluoride (PMSF), 2-carboxy-2′-hydroxy-5′-sulfoformazyl benzene monosodium salt (Zincon, ZI), and trypsin were purchased from Sigma Aldrich. Carbenicillin was purchased from Roth. All remaining chemicals were of analytical grade and were commercially available.

#### The primers used were

gN1F (GCGCGAGCTCGTGCCGATTGATATCGATAAA), gN1R (GCGCAAGCTTCTACAGTTCATCCAGACGGGTA), gN2F (GCGCGAGCTCCAAGAA.

GTTGCACGTCTGC), and gN2R (GCGCAAGCTTCTACTCGTGTTCTTTCATCATTTC).

#### Resins and columns

PD10 desalting and Superdex 75 Increase 10/300 GL columns were purchased from GE Healthcare. Ni-NTA agarose resin was purchased from Qiagen.

### Prediction of IDRs and modeling of chicken nesfatins

The IDRs of gN1 and gN1/2 were predicted with the PONDR VL-XT [29, 30] (available at http://pondr.com) and DynaMine [31, 32] (http://dynamine.ibsquare.be) algorithms based on their amino acid sequences. Structural models were generated using AlphaFold2 via ColabFold [33–35] and visualized with UCSF Chimera [36].

### Expression and purification of recombinant proteins

Plasmids containing cDNA of gN1 and gN1/2 and the preparation of the recombinant proteins were performed as described previously [28]. Briefly, gN1 and gN1/2 sequences were amplified by polymerase chain reaction (PCR) with primers that introduce SacI and BamHI restriction sites, using a modified pQE-80L vector with cloned gNucb2 cDNA as a template [26]. PCR products were then double digested with SacI and BamHI endonucleases (Thermo Scientific) and ligated into the pQE-80L vector using T4 DNA ligase (Thermo Scientific). The resulting vectors were validated by Sanger sequencing (Genomed S.A.). The N-terminus of the proteins contained a 6 × His tag followed by the human rhinovirus 3 C (HRV3C) cleavage site. Following the removal of the 6 × His tag, the peptides contained 4 additional amino acids at the N-terminus preceding the nesfatin sequence.

Proteins were expressed in Bl21 (DE3) pLysS *E. coli* cells (Thermo Scientific). For this purpose, the cells were transformed with 4 ng of each construct, plated onto LB agar supplemented with 50 µg/ml carbenicillin and 35 µg/ml chloramphenicol, and then incubated overnight at 37 °C. Next, selected transformants were transferred to TB (carbenicillin + chloramphenicol) media and grown for 8 h at 37 °C with agitation at 200 rpm. A 30 ml aliquot of the inoculum was added to 0.5 l of fresh TB (carbenicillin + chloramphenicol) medium and grown until the OD600 reached 0.7–0.8. IPTG was then added to a final concentration of 0.25 mM, and the culture was incubated for the next 3 h at 29 °C and 200 rpm. The cells were collected by centrifugation for 8 min at 4 °C and 5,500 g, after which the pellet was suspended in buffer A1 supplemented with 20 µg/ml PMSF. Extracts were stored at -80 °C before use. The extracts were thawed on ice and supplemented with PMSF (20 µg/ml), DNase I (10 µg/ml), and RNase I (10 µg/ml). The cells were then lysed by sonication on ice and incubated for 1 h at 4 °C and 10 rpm on a vertical shaker. After lysing, the mixture was centrifuged (1 h; 4 °C; 18,000 g), and 2 ml (50% v/v) of Ni-NTA resin equilibrated with buffer A1 was added to the supernatant, followed by incubation for 30 min at 4 °C and 10 rpm. The resin was transferred onto a Tricorn column connected to an Äkta Explorer (GE Healthcare) system. Contaminant proteins were eluted with buffer A1 supplemented with either 20 mM (for gN1/2) or 35 mM (for gN1) imidazole. Nesfatins were then eluted with buffer A2. Selected fractions were pooled and desalted using buffer A1 (PD10 columns), followed by overnight incubation with HRV3C protease (Sino Biological) at a 1:1,000 w/w ratio. Afterwards, the solution was incubated for 30 min at 4 °C and 10 rpm with 0.6 ml (50% v/v) of preequilibrated Ni-NTA resin and then loaded onto an empty PD10 column. The flow-through was then concentrated using an Amicon Ultra-4 filter (molecular weight cutoff of 3 kDa) to a final volume of 1 ml. Nesfatins were subsequently further purified using a Superdex 75 Increase 10/300 GL column connected to the Äkta Avant system (GE Healthcare). Separation was monitored at 280 nm, and the concentration was estimated using the following molar extinction coefficients: 4470 1/(M×cm) (for gN1) and 7450 1/(M×cm) (for gN1/2). The extinction coefficients for human nesfatins were determined as described previously [28]. The purity and identity of the proteins were verified by SDS–PAGE (data not shown) and mass spectrometry analysis (Fig. S1). The fully purified protein was quantified at 1.3–2.5 mg (for gN1) and 8–10 mg (for gN1/2) per liter of culture.

### Circular dichroism (CD) spectroscopy

#### Far-UV CD experiments

Far-UV circular dichroism spectra were recorded using a Jasco J-815 spectropolarimeter connected to a Peltier-type temperature controller. Spectra were acquired at 20 °C in 0.1-cm quartz cuvettes at a scanning rate of 50 nm/min from 195 to 260 nm with a bandwidth of 1 nm and 3 accumulations per sample. The protein concentration was 0.15 mg/ml. Each protein sample was suspended in buffer B1 supplemented with either CaCl_2_ (10 mM), EDTA (5 mM), or ZnCl_2_ (20–500 µM). The contribution of the appropriate buffer was subtracted, and then the data were converted to mean residue ellipticity (MRE) and smoothed with a Savitzky–Golay filter (15 points, polynomial order 3). The data from the replicates were averaged. Fractional saturation and free Zn(II) concentration were calculated as described previously [28]. Estimation of the secondary structure content was performed using the BeStSel server with a data range of 200–260 nm [37–39].

#### Thermal denaturation CD experiments

Thermal denaturation profiles were recorded at 208 and 222 nm with the following spectral parameters: a temperature range of 20–90 °C, 2 °C intervals, a ramp rate of 1 °C/min, and a 2 s digital integration time. Proteins (0.25 mg/ml of each) were suspended in buffer B1 supplemented with either 5 mM EDTA or 50–500 µM Zn(II). The obtained data were fitted to either Boltzmann or biphasic dose-response models using OriginPro 2018 software, and the melting temperature (Tm) value was interpreted as the inflection point of the fitted curves.

### Sedimentation velocity analytical ultracentrifugation (SV-AUV)

SV-AUC experiments were performed on Beckman Coulter Proteome Lab XL-I ultracentrifuge (software version 6.0, Beckman Coulter Inc.) equipped with an An-60Ti rotor. The concentrations of gN1 were 1.3, 1.0, and 0.7 mg/ml, and the concentrations of gN1/2 were 1.85, 1.32, and 0.92 mg/ml. The samples were suspended in buffer B1 supplemented with either 5 mM EDTA or 500 µM (for gN1) or 50 µM (for gN1/2) ZnCl_2_. Analysis was conducted at 20 °C and 50,000 rpm. The parameters obtained with SEDNTRP [40] were as follows: the protein partial specific volumes were 0.738 and 0.734 ml/g for gN1 and gN1/2, respectively. The buffer densities were 1.0059 and 1.006 g/ml for buffers supplemented with EDTA and Zn(II), respectively, and the viscosities were 1.0265 and 1.0228 mPa × s for EDTA and Zn(II), respectively. The time-corrected data were analyzed with Sedfit software (version 16.1) with the built-in continuous sedimentation coefficient distribution model *c*(s). The maximum-entropy regularization of the *c*(s) model was set to a confidence level of 0.68 [41, 42].

### Competitive titration of zincon

Competitive titration of Zincon (ZI) was performed using a 1 mM stock of the chromophore in buffer B1. Samples (100 µl each) were prepared in buffer B1 by incubating 50 µM ZI saturated with 50 µM ZnCl_2_ with a range of gN1 concentrations in a 384-well plate (Greiner) for 20 min with shaking. Then, the plate was centrifuged (1,000 g, 2 min, 20 °C) and scanned with a BMG ClarioStar Plus plate reader at 618 nm in absorbance endpoint mode. The obtained data were analyzed according to Kocyła et al. [43] using the previously determined ZI-Zn(II) dissociation constant, *K*_d, ZI_ [28].

### Hydrogen-deuterium exchange coupled with mass spectrometry (HDX-MS)

HDX-MS experiments were performed at the Institute of Biochemistry and Biophysics, Polish Academy of Sciences (IBB PAS), Warsaw, Poland, using a nanoACQUITY UPLC system (Waters) equipped with HDX technology and an HDX manager coupled to a SYNAPT G2 HDMS instrument (Waters). Stocks of N1 and N1/2 (50/100 µM) were prepared in buffer B2 in the presence or absence of 50 µM Zn(II) (for N1/2) or 500 µM Zn(II) (for N1) and incubated after the addition of the ligand for 30 min at 20 °C. The peptide list was generated from nondeuterated samples prepared at 20 °C. This was achieved by adding 5 µl of each protein stock (100 µM) to 45 µl of buffer B2 supplemented (or not) with the appropriate concentration of Zn(II). Then, the samples were mixed with 10 µl of nondeuterated quenching solution S, kept on ice, and immediately frozen in liquid nitrogen. The samples were thawed directly before injection, and the nesfatins were digested online using a 2.1 × 20 mm pepsin column (Poroszyme™, Thermo Scientific) for 1.5 min at 20 °C. After digestion, the peptides were eluted with 0.07% formic acid in water at a flow rate of 200 µl/min onto a reversed-phase ACQUITY BEH C18 VanGuard precolumn, and subsequently onto an ACQUITY UPLC BEH C18 column (Waters) with a 10–35% gradient of acetonitrile in 0.01% formic acid at a flow rate of 90 µl/min and at 0.5 °C. The instrument parameters for MS detection were as follows: ESI in positive mode; capillary voltage, 3 kV; sampling cone voltage, 35 V; extraction cone voltage, 3 V; source temperature, 80 °C; desolvation temperature, 175 °C; and desolvation gas flow, 800 l/h. Peptides were identified using ProteinLynx Global Server Software (Waters).

H/D-exchanged samples were prepared at 5 intervals (10 s, 1 min, 5 min, 30 min, and 2.5 h) using 50 µM protein stocks following the procedure described above. Deuterated buffer B2 (99.99% D_2_O, pH 7.0) supplemented (or not supplemented) with the appropriate concentration of Zn(II) was used. The H/D exchange was quenched with solution S in D_2_O (99.99%, pH 2.3). Additionally, control samples were prepared to assess the minimal and maximal H/D exchange. Samples with minimal H/D exchange were prepared by mixing 10 µl of solution S in D_2_O with deuterated buffer B2 on ice. Then, 5 µl of the protein stock (50 µM) was added, and the samples were immediately frozen in liquid nitrogen. Maximal H/D exchange samples were prepared by incubating 5 µl of the protein stock (50 µM) for 24 h in deuterated buffer B2, followed by quenching with ice-cold solution S. Control samples were prepared in triplicate and analyzed by LC–MS as described above.

The peptide lists derived from nondeuterated samples were used to analyze the exchange data with DynamX 3.0 software (Waters). The PLGS peptide list was filtered by minimum intensity criteria (3,000) and minimal product per amino acid (0.3). All the raw files were processed and analyzed in DynamX 3.0 software. All MS assignments in DynamX were inspected manually. Heatmaps of the exchange differences were exported from DynamX and further data analysis was performed using HaDeX software [44].

### Limited proteolysis

Nesfatins and hNucb2 (at a final concentration of 0.56 mg/ml each in the reaction mixture) were digested for 3.5 h at 20 °C and 350 rpm with sequencing-grade trypsin using a 1:5,000 w/w enzyme-to-protein ratio. Additionally, a 1:1,000 w/w enzyme-to-protein ratio was used for gN1 and hN1. The reactions were performed in buffer B1 supplemented with either 5 mM EDTA (for each nesfatin) or 500 ZnCl_2_ for hN1 and gN1 and 50 µM ZnCl_2_ for gN1/2 and hN1/2. Following the addition of trypsin, 9 µl aliquots were taken from the reaction at specific time intervals and added to the SDS–PAGE loading buffer to stop the reaction. Then, 3 µg of protein was loaded onto an 8–20% homemade polyacrylamide gradient gel, and the digestion products were separated according to Laemmli [45]. To assess the effect of the long incubation time on the digestion products, additional gN1 samples were prepared. One sample set was incubated for 24 h at 20 °C in the presence of either 5 mM EDTA or 500 µM Zn(II) before digestion, while the second set was freshly prepared following the addition of trypsin at a 1:1,000 w/w enzyme-to-protein ratio. Nine-microliter aliquots were taken every 24 h.

### ThT binding studies

#### Digestion of nesfatin-1 in the presence of ThT

Kinetic aggregation studies in the presence of 20 µM ThT were conducted using a BMG Clariostar Plus reader. For this purpose, gN1 and hN1 (30 µM each) were incubated with 5 mM EDTA or 500 µM Zn(II) in the presence or absence of trypsin at a 1:1,000 w/w enzyme-to-protein ratio for 48 h. Each sample (50 µl) was prepared in triplicate on a black 384-well plate (Greiner). The plate was then centrifuged (1,000 g, 2 min, 20 °C) and scanned in fluorescence intensity mode. ThT was excited at 418 nm (20 flashes/well), and fluorescence emission was measured at 490 nm. The data are expressed as normalized fluorescence units (NFUs) calculated by dividing the number of raw fluorescence units (RFUs) of the samples by the mean free fluorophore emission.

#### ThT binding of nesfatins titrated with zn(II)

The binding of ThT (5 µM) to nesfatins (10 µM each), titrated with Zn(II) was monitored on a BMG Clariostar plate reader by excitation of the fluorophore at 418 nm (20 flashes/well) and scanning over emission in the 440–600 nm range. Each sample (50 µl) was prepared in triplicate on a 384-well plate, which was centrifuged (1,000 g, 2 min, 20 °C) before analysis. The spectra were then smoothed in OriginPro software using a Savitzky–Golay filter (15 points, polynomial order 2). The RFU at the emission maximum (483 nm and 476 nm for nesfatin-1 and nesfatin-1/2, respectively) versus Zn(II) concentration was then fitted to a Hill1 model using OriginPro software.

## Results

### The structures of chicken nesfatins are predicted to be disordered and context sensitive

**Fig. 2:**
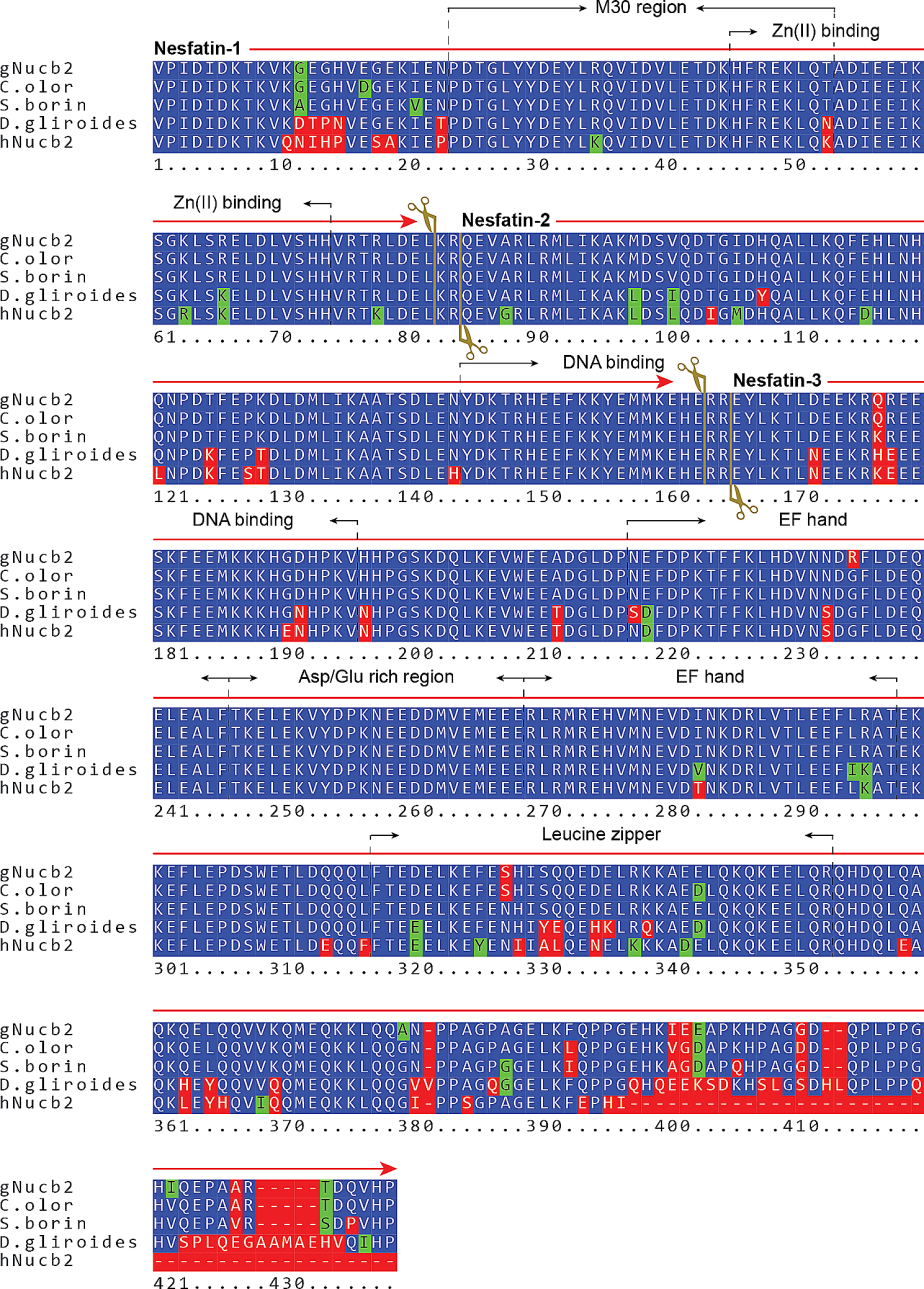
Chosen BLAST hit sequences aligned to the gNucb2 sequence (top of the panel). Green represents similar residues, red represents different residues, and blue represents identical residues. The PCs cleavage sites are marked with dark yellow scissors and lines. The nesfatin-1, nesfatin-2, nesfatin-3, M30 region and Zn(II) binding sequence span are marked at the top of the alignment rows. Alignments were made with the pyBoxshade package

The sequences of amino acids in nesfatin-1 and nesfatin-1/2 are highly conserved between chickens and humans, showing 87% and 85% similarity among N1 and N1/2 homologs, respectively. Notably, there is a significant cluster of differing amino acid residues at the N-terminus of both gN1 and hN1, particularly between residues 11–19. Additionally, singular substitutions, predominantly involving similar amino acid residues, are observed elsewhere in the sequences (Fig. 2). In contrast, while the amino acid sequences of chicken and human N2 homologs display singular substitutions involving similar aa residues at the N-terminus, a different pattern emerges for the C-terminus. (Fig. 2). The structures of the gN1, gN2, and gN1/2 models (Fig. 1B) generated with the AlphaFold2 algorithm using ColabFold [33–35] appear to be mostly *α*-helical, with a putative IDR between 1 and 30 aa residues. Moreover, the structure of gN1/2 was predicted as the sum of the isolated fragments, which, based on the results published in this paper, was proven not to be the case (see below). The AlphaFold2 helical models of gN1, gN2, and gN1/2 also differed from the results obtained with the PONDR VL-XT [29, 30] and DynaMine algorithms [31, 32]. According to the PONDR VL-XT algorithm, three segments within the gN1 sequence (depicted in Fig. 1C) were predicted to be disordered: residues 1–9, 52–74, and 84–86. Collectively, these regions comprise almost 41% of the gN1 structure. Additionally, analysis of the gN1 sequence using the DynaMine algorithm (Fig. 1D) revealed that the region spanning residues 32–62 is predicted to be rigid, whereas the remaining structure of gN1 appears to be context dependent. Sequence analysis of gN1/2 revealed three regions predicted to be disordered by the PONDR VL-XT algorithm (Fig. 1E): residues 1–9, 52–101, and 165–167. Additionally, two regions were identified as ordered: 10–51 and 102–164. Overall, approximately 36% of the gN1/2 structure is expected to be disordered. Furthermore, the DynaMine results (Fig. 1F) indicated the presence of two rigid regions within the gN1/2 structure: residues 33–62 and 79–99. The remaining parts of the gN1/2 structure were predicted to be context dependent.

Consequently, the outcomes of PONDR VL-XT and DynaMine predictions for both gN1 and gN1/2 structures suggest that rather than being fully structured, both structures exhibit a mosaic-like pattern and are context dependent.

### Zn(II) exerts opposite effects on the secondary structure of nesfatins

#### Apo-gN1 and apo-gN1/2 are members of the IDP family

**Fig. 3:**
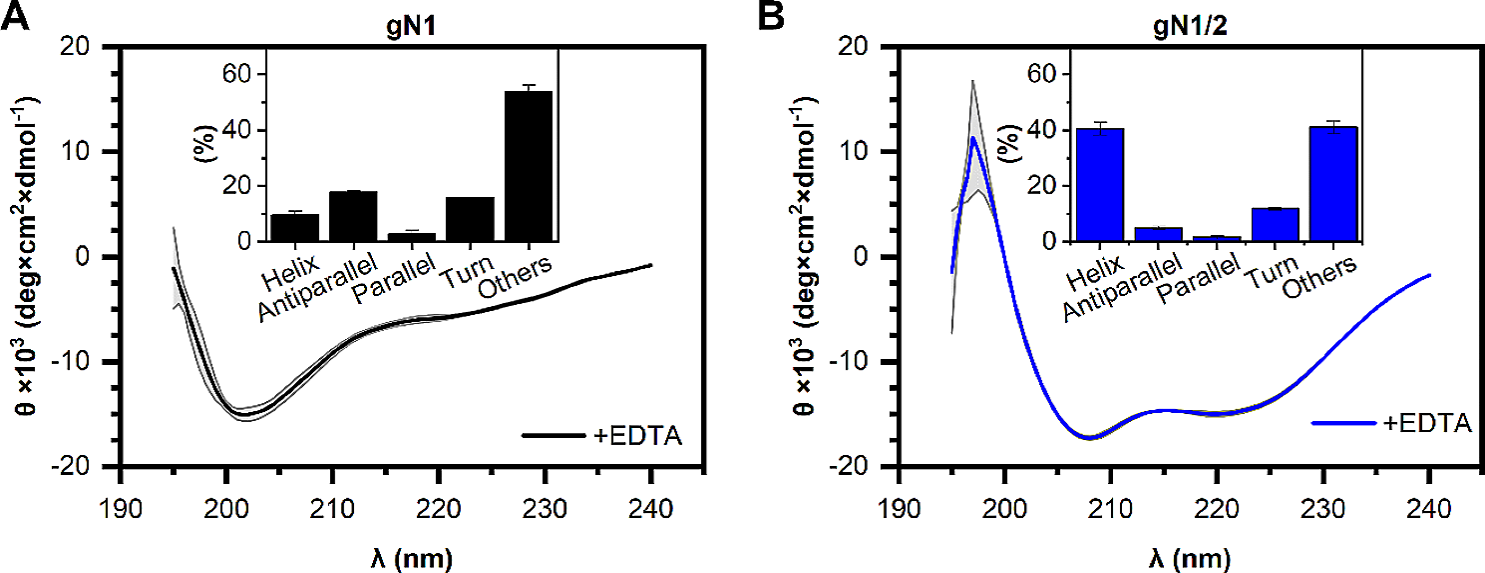
Average CD spectra of (**A**) gN1 and (**B**) gN1/2 (0.15 mg/ml each) in the presence of 5 mM EDTA. The grey area represents the standard deviation. The inset graph shows the secondary structure content calculated with BeStSel

To validate the predictions regarding the intrinsic disorder propensity of gN1 and gN1/2, CD experiments were conducted. The resulting CD spectra of apo-gN1 and apo-gN1/2 are depicted in Fig. 3. In the gN1 spectrum (Fig. 3A), a characteristic negative maximum at 201 nm was observed, indicating a high content of IDRs [46]. Furthermore, analysis using a double-wavelength plot (Fig. S2) suggested that gN1 was situated closer to the cluster associated with coil-like IDPs, which is indicative of a highly dynamic structure. In contrast, the spectrum of gN1/2 exhibited two negative maxima at 208 and 222 nm (Fig. 3B), suggesting a significant contribution of α-helices to the structure [46]. Indeed, deconvolution of the spectra using the BeStSel server revealed that 54 ± 2% of the gN1 secondary structure is disordered (Table 1). Notably, there were antiparallel β-sheets (18.0 ± 0.4%) and turns (16.0 ± 0.2%) in the gN1 structure. Conversely, the deconvolution of gN1/2 spectra indicated a high content of α-helices (40 ± 2%), along with significant amounts of IDRs (41 ± 2%) and turns (12 ± 2%). Therefore, the secondary structure of gN1 appears to be predominantly disordered, while gN1/2 exhibits a mosaic-like structure with intertwined disordered and ordered regions. These findings are consistent with the results of the IDR predictions (see Sect. 2.1).

**Table 1:** Secondary structure content of chicken nesfatins in the presence of 5 mM EDTA estimated with BeStSel

Protein	α-Helix (%)	Antiparallel (%)	Parallel (%)	Turn (%)	Other (%)
gN1	9 ± 2	18.0 ± 0.4	3 ± 1	16.0 ± 0.2	54 ± 2
gN1/2	40 ± 2	5.0 ± 0.6	2.0 ± 0.5	12.0 ± 0.4	41 ± 2

The data represent the average ± SD from 3 measurements

#### Zn(II) sensing is conserved between chicken and human homologs of nesfatin

**Fig. 4:**
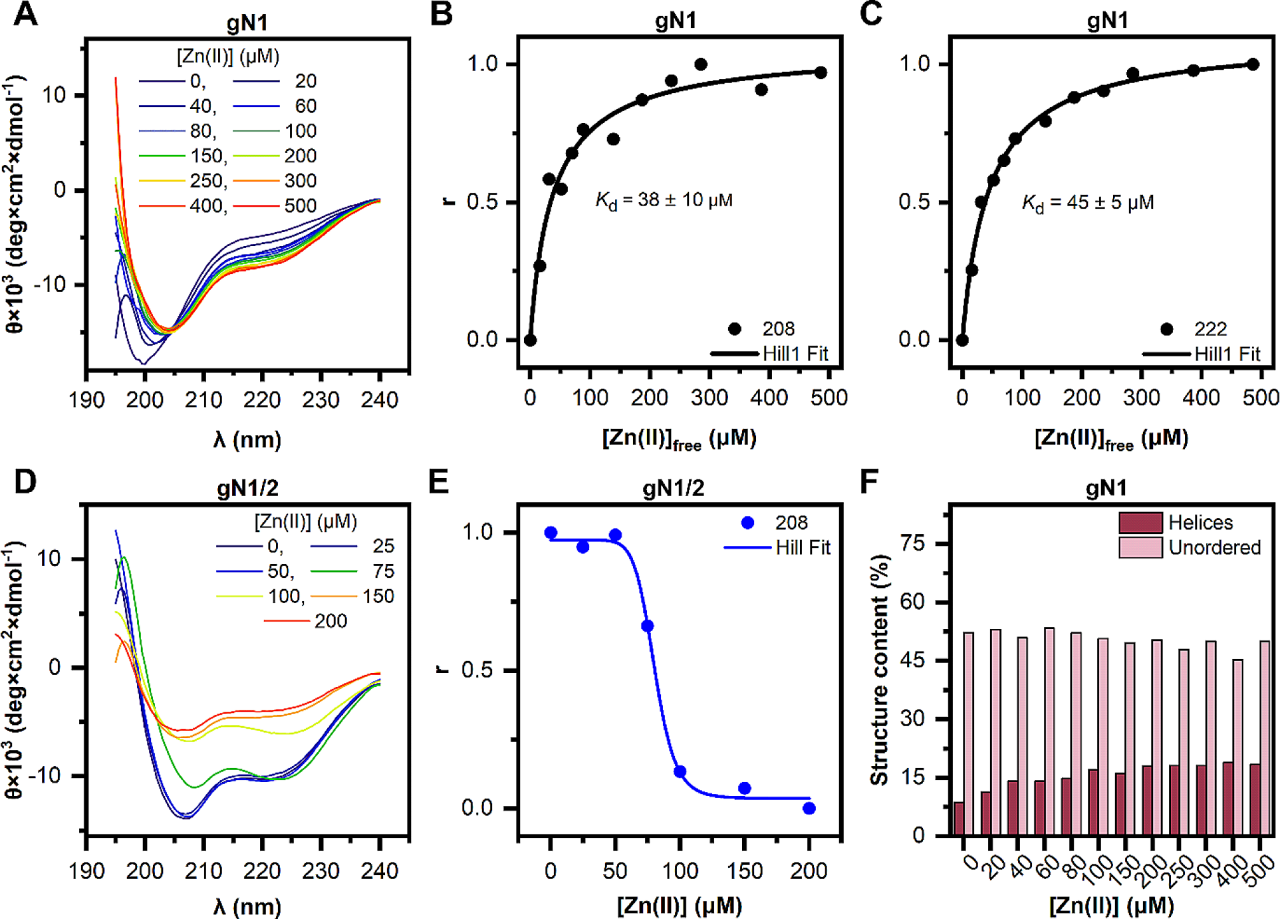
Changes in the CD spectra of chicken nesfatins titrated with Zn(II). (**A**, **D**) CD spectra of gN1 and gN1/2 over a range of Zn(II) concentrations. Fractional saturation (r) of gN1 with Zn(II) and the Hill fit of the data at 208 (**B**) and 222 nm (**C**). (**E**) Relative abundance of gN1/2 in the presence of Zn(II) ions and the Hill fit of the data. (**F**) Changes in the helical (burgundy) and unordered (light pink) structural content of gN1 titrated with Zn(II) (estimated with BeStSel)

In the subsequent step, we investigated the impact of Ca(II) and Zn(II) on the secondary structure of chicken nesfatins. No alterations were observed in the CD spectra of gN1 and gN1/2 in the presence of Ca(II) (Fig. S3). However, Zn(II) had a pronounced effect on both the gN1 and gN1/2 peptides. A substantial Zn(II) concentration-dependent redshift of the negative maximum at 201 nm to 208 nm was noted, accompanied by a deepening of the negative maximum at 222 nm in the CD spectra of gN1 (Fig. 4A). This shift indicates a disorder-to-order transition, corroborated by the deconvolution results obtained using BeStSel (Table 2). Notably, there was a 2.3-fold increase in the α-helical content from 8 to 18.3%, along with a decreasing trend in the content of IDRs from 53 to 45.2% (Fig. 4F). Additionally, an isosbestic point at approximately 204 nm was observed. The data obtained at 208 nm (Fig. 4B) and 222 nm (Fig. 4C) versus the free Zn(II) concentration were fitted to the Hill equation, yielding *K*_d_ values of 38 ± 10 µM and 45 ± 5 µM, respectively.

**Table 2:** Changes in the secondary structure content of gN1 in the presence of Zn(II)

[Zn(II)] (µM)	α-Helix (%)	Antiparallel (%)	Parallel (%)	Turn (%)	Other (%)
0	8.6	21.4	0.0	18.0	52.0
20	11.3	18.9	0.0	16.8	53.0
40	14.0	18.4	0.4	16.4	50.8
60	14.0	16.1	1.5	15.1	53.3
80	14.7	16.4	2.7	14.2	52.0
100	17.0	15.1	2.8	14.5	50.6
150	16.0	16.0	3.2	15.3	49.5
200	17.9	14.7	2.4	14.7	50.3
250	18.1	16.3	1.8	15.9	47.9
300	18.1	15.1	2.5	14.5	49.9
400	18.9	16.6	4.4	14.9	45.2
500	18.3	14.5	2.8	14.4	50.0

Compared with that of gN1, the binding of Zn(II) by gN1/2 elicited a markedly different response. At low Zn(II) concentrations, there was only a subtle effect on the CD spectra of gN1/2 until reaching the threshold of 50 µM (Fig. 4D). Subsequently, a Zn(II) concentration-dependent reduction in the CD signal was observed, which was attributed to the precipitation and/or aggregation of the peptide. This process appeared to be cooperative, as evidenced by the sigmoidal shape of the curve fitted to the CD data at 208 nm (Fig. 4E). The concentration of Zn(II) resulting in a 50% decrease in the CD signal was estimated to be 81 ± 2 µM from the fitting.

In summary, Zn(II) exerted contrasting effects on gN1 and gN1/2, facilitating the structurization of the former while causing significant destabilization of the latter. Furthermore, this phenomenon was consistently observed in all the experiments conducted in this study (see below).

#### Binding of Zn(II) destabilizes the structure of chicken and human nesfatin homologs

**Fig. 5:**
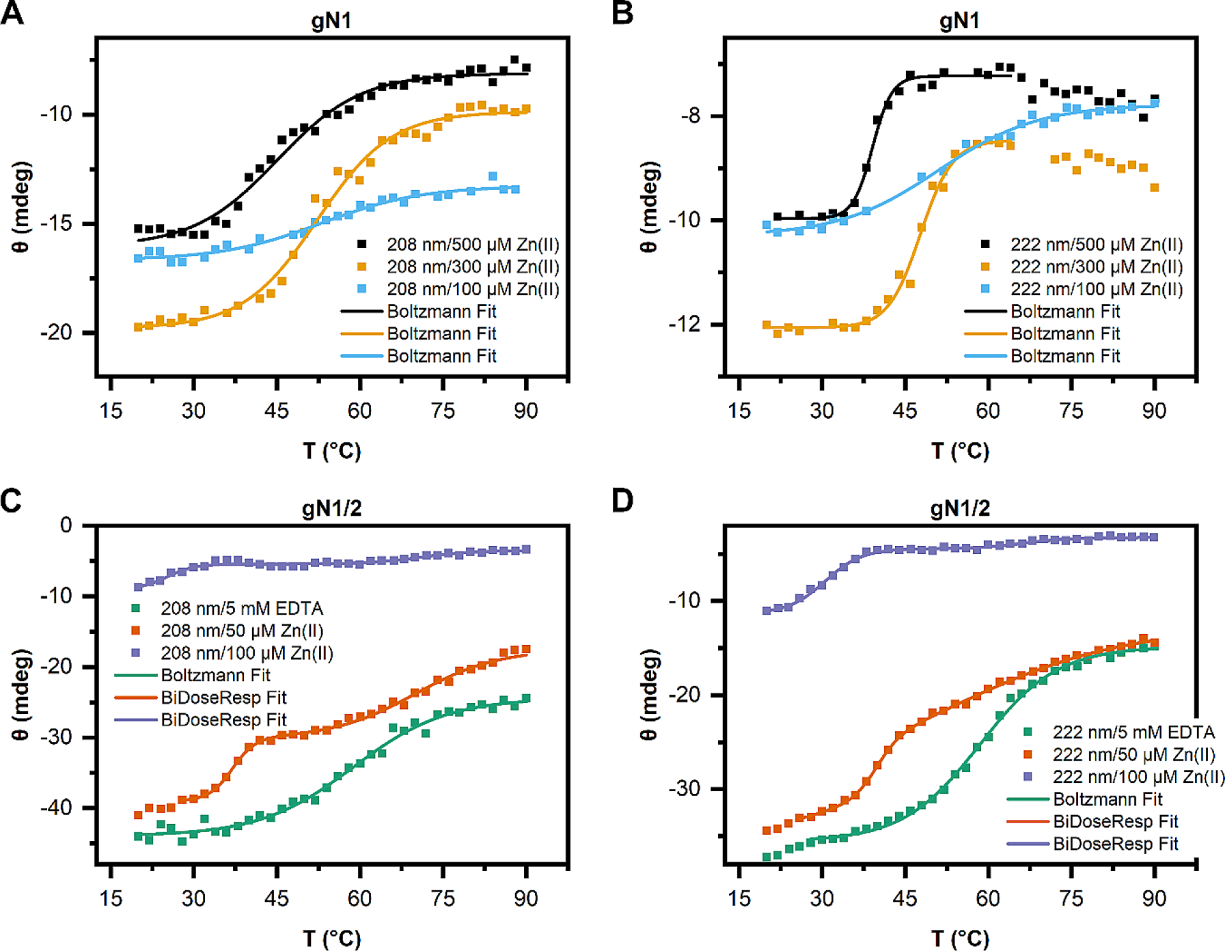
Thermal denaturation curves measured by CD at 208 (**A**, **C**) and 222 nm (**B**, **D**) in the presence of either EDTA or Zn(II) at the concentrations specified in the legend. (**A**, **B**) Thermal denaturation profile of gN1. (**C**, **D**) Thermal denaturation profile of gN1/2

To investigate the effects of Zn(II) binding by chicken and human nesfatins on peptide stability, we conducted thermal CD experiments. The denaturation curves of chicken and human homologs of apo-N1 exhibited no defined melting temperature (Tm), as expected for members of the IDP family (data not shown). However, the Zn(II) concentration-dependent formation of ordered structures and oligomerization under Zn(II) treatment (see Sect. 2.2.2 and Sect. 2.3) enabled the observation of defined Tm values for both homologs. The thermal denaturation profiles of holo-gN1 recorded at 208 and 222 nm (Fig. 5A and B) displayed sigmoidal denaturation curves and a Zn(II) concentration-dependent decrease in the Tm from 53 °C to 45–39 °C (Table 3). Similarly, a decrease in the Tm was observed for gN1/2 (Fig. 5C and D), as the presence of Zn(II) resulted in a significant decline of the Tm value from 58 °C in the absence of ions (5 mM EDTA) to 29–25 °C (Table 3) in the presence of Zn(II), similar to that of gN1. Interestingly, the thermal denaturation profiles of holo-gN1/2 at 208 and 222 nm exhibited biphasic characteristics in the presence of Zn(II) (Fig. 5C and D; dark orange and purple curves). The first Tm oscillated between 25 and 40 °C, while the second estimated Tm ranged between 53 and 71 °C. These results underscore the dual nature of this protein, which is conserved among chicken and human homologs (see below).

**Table 3:** Thermal stability of nesfatins in the presence/absence of Zn(II) measured by CD spectroscopy

Protein	Chemical	Tm at 208 nm [°C]	Tm at 222 nm [°C]
gN1	(100 µM) Zn(II)	53 ± 1	51 ± 1
	(300 µM) Zn(II)	52.3 ± 0.6	47.7 ± 0.4
	(500 µM) Zn(II)	45.1 ± 0.9	39.1 ± 0.3
gN1/2	(5 mM EDTA)	58.8 ± 0.7	58.5 ± 0.3
	(50 µM) Zn(II)	37.0 ± 0.4	39.8 ± 0.3
		69.9 ± 0.7	53 ± 4
	(100 µM) Zn(II)	25 ± 1	30.5 ± 0.3
		71 ± 2	66 ± 2
hN1	(100 µM) Zn(II)	59 ± 1	48.1 ± 0.9
	(300 µM) Zn(II)	41.2 ± 0.9	39.6 ± 0.2
	(500 µM) Zn(II)	33.6 ± 0.8	33.1 ± 0.2
hN1/2	(5 mM EDTA)	61 ± 1	59.0 ± 0.3
	(50 µM) Zn(II)	31.8 ± 0.2	36.2 ± 0.1
		74 ± 2	71 ± 2
	(100 µM) Zn(II)	–	35 ± 2
		–	55 ± 0.9

**Fig. 6:**
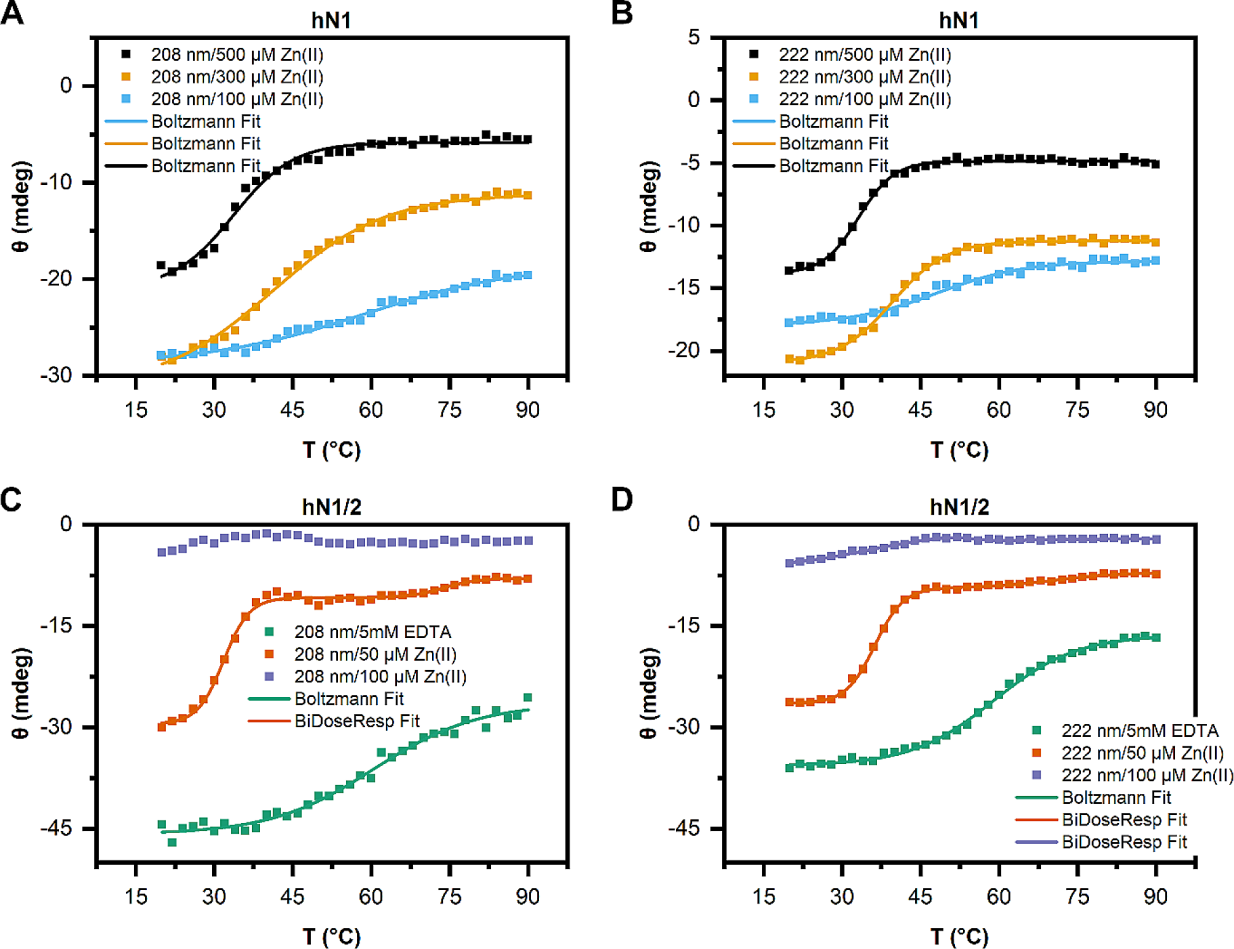
Thermal denaturation curves measured by CD at 208 (**A**, **C**) and 222 nm (**B**, **D**) in the presence of either EDTA or Zn(II) at the concentrations specified in the legends. (**A**, **B**) Thermal denaturation profile of hN1. (**C**, **D**) Thermal denaturation profile of hN1/2

The denaturation of human nesfatins yielded results comparable to those described above for chicken homologs. Consequently, a Zn(II)-concentration-dependent decrease in the Tm was observed for both hN1 (Fig. 6A and B) and hN1/2 (Fig. 6C and D). The denaturation profile of apo-hN1 exhibited no discernible Tm (data not shown), while the profiles of holo-hN1 were sigmoidal at both wavelengths, with a decrease in the Tm value from 59 to 33 °C (Table 3) in response to Zn(II) treatment. These values corresponded well with the Tm values of gN1 in the presence/absence of Zn(II). The denaturation curve of hN1/2 in the absence of ions (5 mM EDTA) was sigmoidal at 208 and 222 nm (Fig. 6C and D; green curves) with estimated Tm values of 61 and 59 °C, respectively. Zn(II) treatment induced a decrease in the Tm value to 36 °C (Table 3). Biphasic denaturation profiles were also observed for hN1/2. The second Tm was estimated to be approximately 71–74 °C (Fig. 6C and D; orange curves) at 50 µM Zn(II) and 55 °C at 100 µM Zn(II) (Fig. 6D, purple curve).

Consequently, the decrease in Tm for all homologs indicated the destabilization of their structures under Zn(II) treatment. However, based on the results of CD experiments (see Sect. 2.2.2), the decrease in Tm value for N1 homologs was attributed to their gradual structurization caused by Zn(II), while for N1/2 homologs, it was a result of their instability in the presence of Zn(II). Thus, the effects of Zn(II) on gN1 and gN1/2 were once again emphasized.

### Zn(II) induces the oligomerization and/or aggregation of chicken nesfatins

**Fig. 7:**
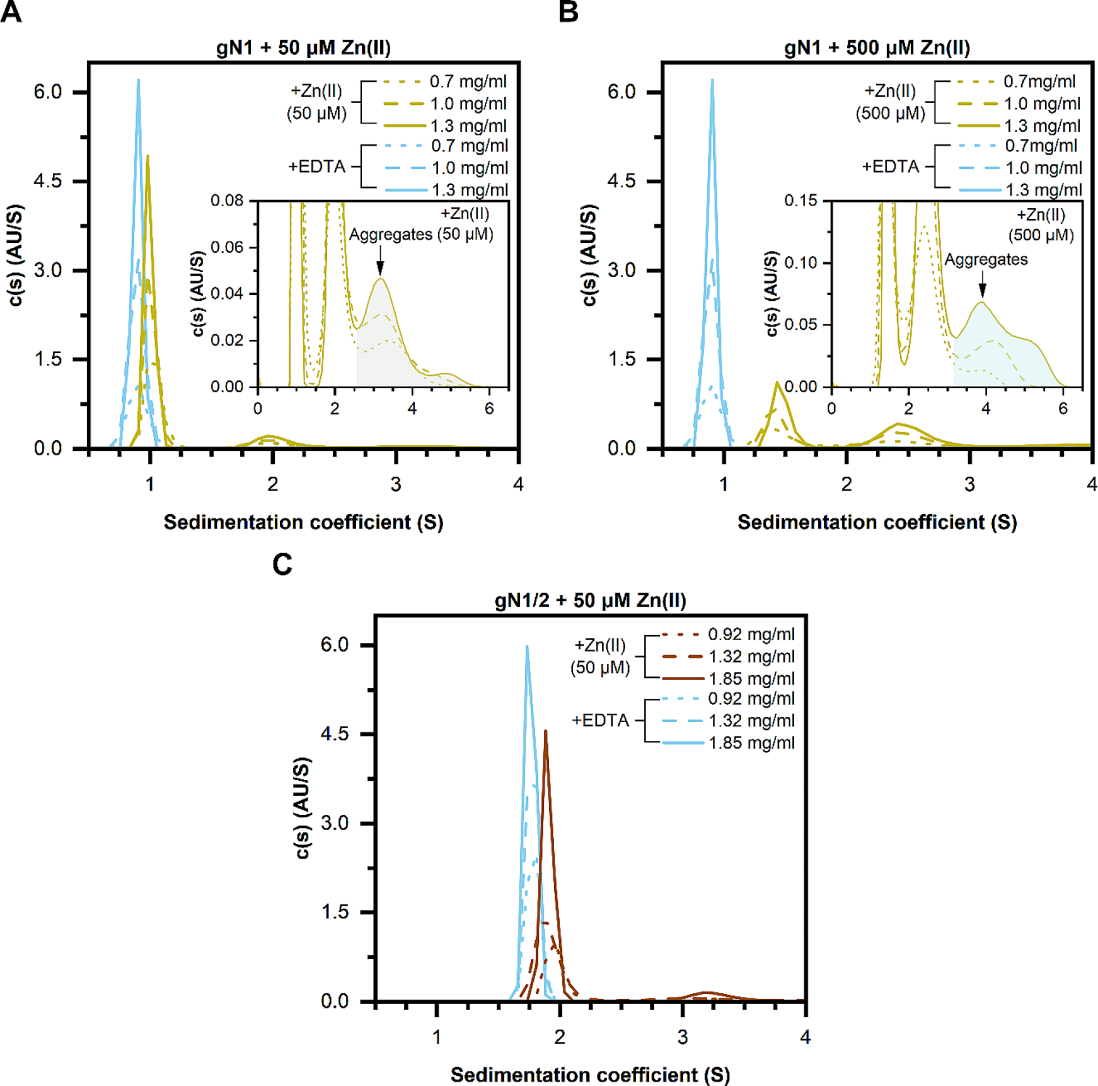
SV-AUC analysis of chicken nesfatins in the presence of 5 mM EDTA (light blue lines) and 50/500 µM Zn(II) (**A**, **B**) distribution plot of gN1 (mustard lines) and (**C**) gN1/2 (brown lines) at the protein concentrations specified in each panel. The inset graphs are enlarged on the scale of the + Zn(II) samples in the region containing aggregates

The tertiary structure and possible formation of quaternary structures were analyzed using sedimentation velocity analytical ultracentrifugation (SV-AUC) experiments. In the absence of ions, both gN1 (Fig. 7A and B) and gN1/2 (Fig. 7C) exhibited a single peak with a very narrow and spike-like continuous sedimentation coefficient distribution *c*(s). Under these conditions, the s_(20,w)_ parameter oscillated approximately 0.94 and 1.86 S for gN1 and gN1/2, respectively (Table 4). The calculated apparent molecular weights (M_app_) were approximately 10 kDa and 21 kDa for gN1 and gN1/2, respectively. These values corresponded very well to the theoretical values and values derived from mass spectrometry experiments (see Fig. S1). Additionally, the f/f_0_ coefficient values of apo-gN1 and apo-gN1/2 were approximately 1.8 and 1.5, respectively.

In the presence of Zn(II), both gN1 and gN1/2 exhibited significant changes in their *c*(s) distributions compared to those in the presence of 5 mM EDTA. There was a concentration-dependent shift in the s_(20,w)_ parameter of gN1 from 0.94 S (100% relative abundance; RA) to 1.08 (83% RA, Table S1) and 1.52 S (56% RA, Table 4) in the presence of 50 and 500 µM Zn(II), respectively. Simultaneously, a second population of sedimenting species emerged at approximately 2.16 (17% RA) and 2.6 S (44% RA) with M_app_ values of 33 and 28 kDa in the presence of 50 and 500 µM Zn(II), respectively. These changes were accompanied by a strong decrease in the f/f_0_ parameter from 1.75 to 1.22, indicating a major compaction of the gN1 structure. In contrast to previously studied hN1 [28], some aggregation of gN1 was observed, as evidenced by the increase in the third peak at approximately 3.5 and 4–4.5 S (inset graphs of Fig. 7A and B, respectively) in the presence of 50 and 500 µM Zn(II), respectively. Additionally, changes were observed in the *c*(s) distribution of gN1/2 (Fig. 7C). There was an increase in the s_(20,w)_ parameter from 1.86 (100% RA) to 2.05 S (87% RA) in the presence of 50 µM Zn(II). Furthermore, a new population of sedimenting molecules represented by a broad peak at approximately 3.5 S (13% RA) and an M_app_ of 44 kDa emerged, indicating the formation of a dimer. A decrease in the f/f_0_ parameter from 1.5 to 1.3 was also observed, which was associated with the compaction of the gN1/2 structure.

**Table 4:** Hydrodynamic properties of chicken nesfatins

Protein	c [mg/ml]	Compound	rmsd ×10^3^	s_(20, w)_ [S]	f/f_0_	*R*_h_ [nm]	M_app_ [kDa] (%)
gN1	0.7	(5 mM) EDTA	7.97	0.94	1.75	2.53	10.3 (100)
	1.0		9.10	0.94	1.75	2.53	10.2 (100)
	1.3		9.39	0.94	1.75	2.52	10.2 (100)
	0.7	(500 µM) Zn(II)	8.16	1.52	1.22	1.88	12.3 (56)
				2.60		2.46	27.6 (44)
	1.0		8.75	1.50	1.25	1.92	12.4 (49)
				2.59		2.52	28.1 (51)
	1.3		9.42	1.54	1.21	1.86	12.4 (49)
				2.63		2.43	27.6 (51)
gN1/2	0.92	(5 mM) EDTA	8.24	1.87	1.47	2.70	21 (100)
	1.32		8.94	1.86	1.45	2.65	21 (100)
	1.85		9.89	1.85	1.45	2.65	21 (100)
	0.92	(50 µM) Zn(II)	7.92	2.05	1.24	2.20	19 (87)
				3.49		2.87	43 (13)
	1.32		8.36	2.00	1.31	2.35	20 (85)
				3.64		3.05	44 (15)
	1.85		8.98	2.00	1.30	2.32	20 (87)
				3.43		3.04	44 (13)

The numbers in the round brackets represent the percentage of each fraction relative to the main sedimenting species (100%)

Taken together, these results highlight the conservation of Zn(II) sensing ability between chicken and human nesfatin homologs. However, chicken nesfatins appeared to be more prone to aggregation than human nesfatins in response to Zn(II), indicating differences in the interaction outcomes between the homologs.

### gN1, but not gN1/2, competes for Zn(II) with ZI

**Fig. 8:**
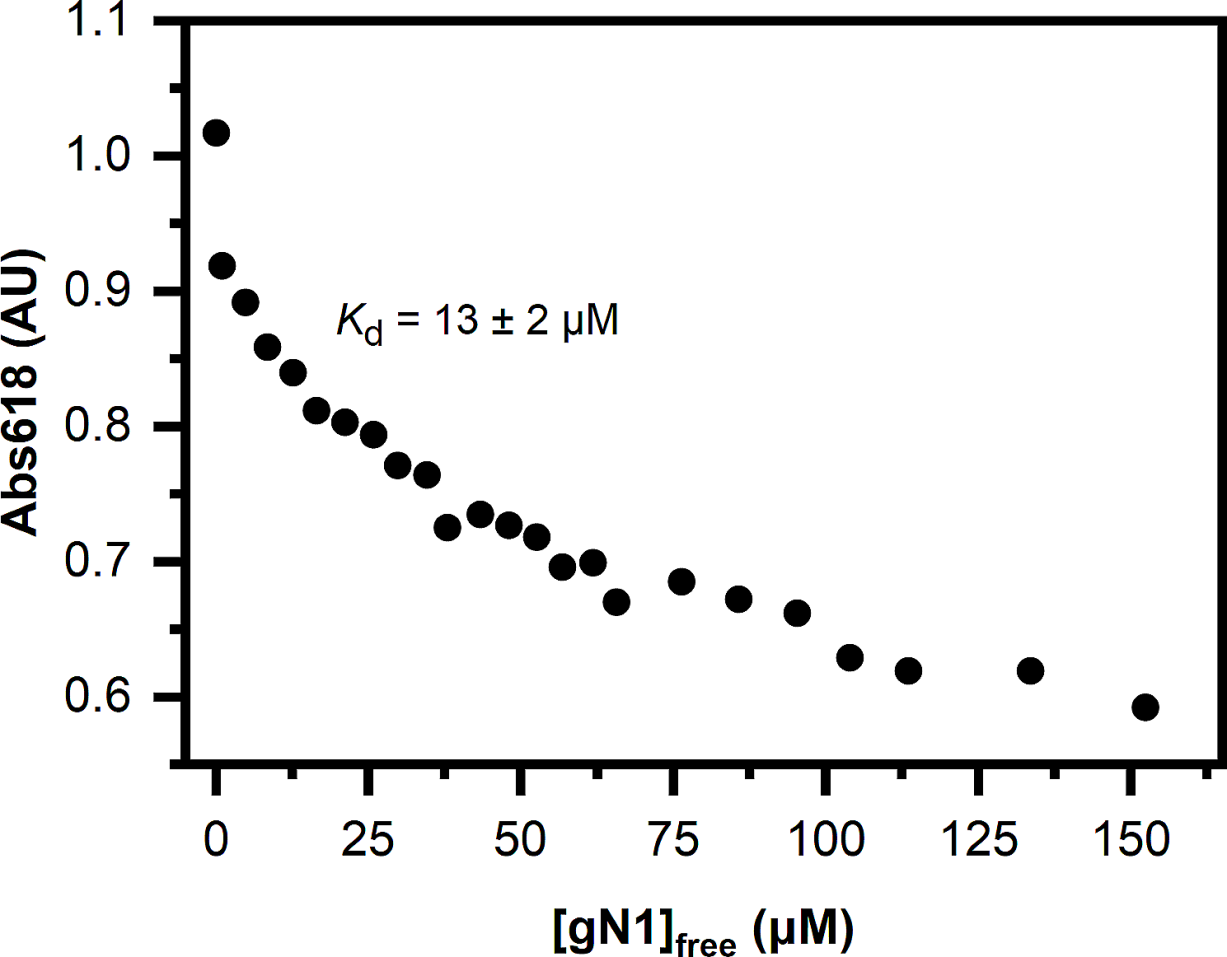
Competitive titration of ZI (50 µM) with gN1

To further investigate the Zn(II) binding capability of chicken nesfatins, we examined whether they could compete for the ion with Zincon (ZI). Upon treatment with gN1 (Fig. 8), the absorbance at 618 nm (Abs618) decreased in a concentration-dependent manner. The apparent *K*_d_ calculated according to Kocyła et al. [43] was estimated to be 13 ± 2 µM. This value is consistent with the results obtained for hN1 previously [28]. However, it differed from the values obtained by other methods described here (see Sect. 2.2.2 and 2.7). This disparity could be attributed to differences in the stoichiometry of the binding and/or the interaction of the peptide with the chromophore. Surprisingly, we observed no change in Abs618 of ZI-Zn(II) titrated with gN1/2 (data not shown). In contrast, we previously demonstrated that hN1/2 was able to compete for Zn(II) with ZI [28]. The inability of gN1/2 to compete for Zn(II) with ZI could partly stem from the fact that the Zn(II) binding region of gN1/2 appears to be more protected than that of hN1/2 (see Sect. 2.5). This finding underscores differences in the mechanism and/or effects of Zn(II) binding to the peptides between the homologs.

In summary, the above results support the conservation of the ability of gN1 and hN1 to compete with ZI for Zn(II) binding, thereby emphasizing differences between the interactions of gN1/2 and hN1/2 homologs with Zn(II).

### Zn(II) exerts Janus effects on chicken nesfatins via the structurization of the M30 region of gN1 and its exposure alongside two additional regions in gN1/2

To map Zn(II) binding regions and/or changes in the solvent exposure of the nesfatin backbone under Zn(II) treatment and to compare them with those found in the full-length protein, hydrogen-deuterium exchange mass spectrometry (HDX-MS) experiments were performed.

**Fig. 9:**
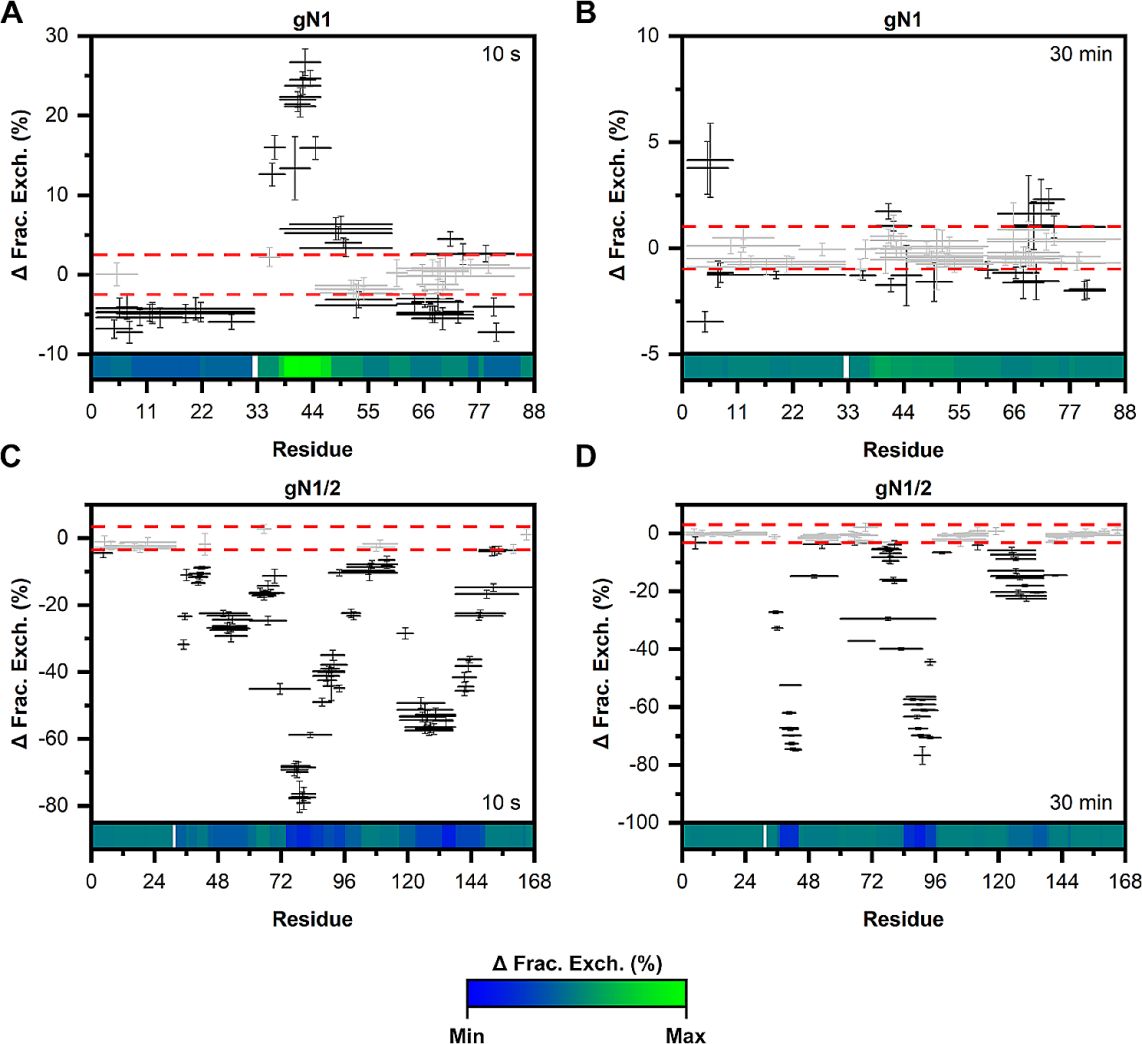
HDX-MS analysis of gN1 (**A**, **B**) and gN1/2 (**C**, **D**). The data represent the difference in the fractional exchange between the -Zn(II) and + Zn(II) states (Woods plot) at the intervals specified in each panel. Statistically significant and insignificant exchanges are depicted as solid black and gray lines, respectively. The confidence threshold of 0.98 is marked by the dashed red lines. The heatmap at the bottom of each panel is colored (with the gradient shown at the bottom of the figure) according to the data range (from every exposure time) of each individual protein

**Fig. 10:**
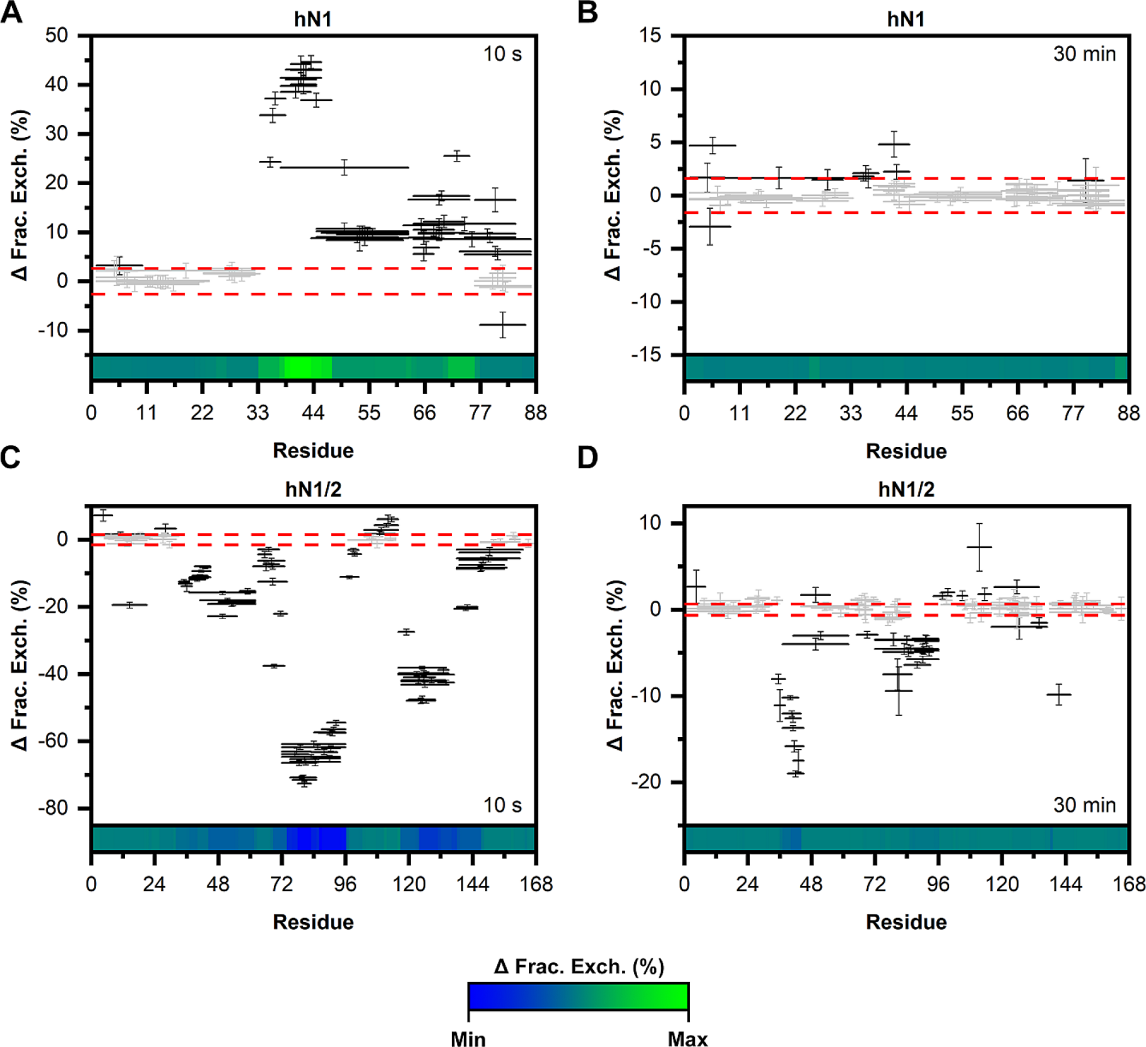
HDX-MS analysis of hN1 (**A**, **B**) and hN1/2 (**C**, **D**). The data represent the difference in the fractional exchange between the -Zn(II) and + Zn(II) states at the intervals specified in each panel. Statistically significant and insignificant exchanges are depicted as solid black and gray lines, respectively. The confidence threshold of 0.98 is marked by the dashed red lines. The heatmap at the bottom of each panel is colored (with the gradient shown at the bottom of the figure) according to the data range (from every exposure time) of each individual protein

Before conducting H/D exchange, peptide maps were generated for each peptide to achieve the best sequence coverage. This was accomplished by the online digestion of apo- and holo-nesfatins using a pepsin column. The peptide maps obtained from the -Zn(II) and + Zn(II) conditions were then combined, resulting in 68 peptides for gN1 (100% sequence coverage, 10.3 redundancy), 96 peptides for gN1/2 (100%, 7.47), 70 peptides for hN1 (100%, 9.5), and 106 peptides for hN1/2 (100%, 7.87). H/D exchange was monitored in the 10 –150 min time range. After 10 s, apo-gN1 (Fig. S4A, B, C) and apo-hN1 (Fig. S5A, B, C) showed almost complete exchange, which was excepted since both proteins are completely disordered. However, under Zn(II) treatment, there was a statistically significant decrease in the fraction exchange difference in the Woods plots of holo-gN1 (Fig. 9A) and holo-hN1 (Fig. 10A) after 10 s of exposure in the 33–47 aa and 33–55 aa regions, respectively. This is attributed to the disorder-to-order transition and putative oligomerization of both neuropeptides (see Sect. 2.3). After 30 min of exposure, holo-gN1 (Fig. 9B) and holo-hN1 (Fig. 10B) were completely exchanged, as there was almost no significant difference between the -/+ Zn(II) states.

The H/D exchange patterns of apo-gN1/2 (Fig. S4D, E, F) and apo-hN1/2 (Fig. S5D, E, F) exhibited a distinct contrast to the results described above for N1 homologs. Specifically, both N1/2 homologs contained intertwined regions characterized by either rapid or slow H/D exchange. For gN1/2, the regions spanning 1–32, 97–115, and 138–167 aa showed almost complete exchange within 10 s. Conversely, the regions spanning 33–96 and 116–137 aa were significantly protected against exchange. Notably, the regions spanning 33–45 and 60–96 aa, especially the peptides covering 84–96 aa, were not fully exchanged even after 150 min (Fig. S4F), indicating their involvement in the structure-forming core. Similarly, apo-hN1/2 also contained three regions characterized by fast H/D exchange: 1–32, 97–116, and 138–167 aa. Conversely, slow exchange to deuterons was observed in the regions spanning 33–96 and 116–137 aa, particularly in the peptides spanning 38–45 and 65–96 aa, which were not completely exchanged for up to 30 min (Fig. S4D). This finding underscores the particularly good conservation of IDRs and structured regions between the homologs. Moreover, these results are in very good agreement with the results obtained previously for the apo-forms of both homologs [26, 27].

Surprisingly, significant and unexpected differences in the patterns of H/D exchange occurred in the presence of Zn(II) for both N1/2 homologs. Particularly, noteworthy was the substantial negative difference in fractional H/D exchange between the -/+ Zn(II) states for gN1/2 and hN1/2. For gN1/2, the peptides covering 33–64, 72–102, and 116–148 aa exhibited a moderate to strong increase in H/D exchange compared to the EDTA-containing sample after 10 s of exposure (Fig. 9C). Furthermore, after 30 min, the Zn(II)-induced increase in isotope exchange remained prominent (Fig. 9D). Notably, peptides encompassing 33–45, 60–96, and 117–137 aa still exhibited significant differences in exchange between the -/+ Zn(II) states. Similarly, for Zn(II)-hN1/2, the regions spanning 33–62, 72–96, and 116–137 aa showed a moderate to very substantial increase in H/D exchange compared to the EDTA-containing sample at the 10 s exposure time (Fig. 10C). This Zn(II)-induced acceleration of exchange remained highly visible after 30 min (Fig. 10D), particularly for the peptides encompassing 34–45 and 76–93 aa. The observed increase in H/D exchange in these regions of gN1/2 and hN1/2 aligns with the results obtained previously for full-length homologs [27]. However, it is worth noting that the region spanning 34–45 aa in holo-hN1/2 remains fairly protected against H/D exchange, particularly when compared to holo-gN1/2.

Thus, Zn(II) exhibited a Janus effect on the M30 region of chicken and human homologs of N1 and N1/2. Zn(II)-driven structurization and putative oligomerization of gN1 and hN1 resulted in the concealment of their bioactive M30 core, suggesting that this might influence their anorexigenic effect [6]. On the other hand, the exposure of the M30 region and the region recognized by the PCs in gN1/2 and hN1/2 under Zn(II) treatment (which were greatly protected in the apo-state) suggested that the presence of the ion might be critical for enabling the processing of the precursor.

### Zn(II) binding protects N1 against proteolysis but facilitates proteolysis in N1/2 homologs

To further investigate and visualize the effects of Zn(II) binding on N1 and N1/2 homolog structures, especially in the context of HDX-MS results (see Sect. 2.5), limited proteolysis experiments were conducted. These reactions were carried out in the absence and presence of Zn(II) using trypsin, which cleaves at the C-terminus of the Arg and Lys amino acid residues [47]. Typically, the globular domains of proteins tend to be poor substrates for proteases under physiological conditions, while well-exposed unstructured regions are considered good substrates [48].

Surprisingly, apo-gN1 (Fig. S6A) and apo-hN1 (Fig. S7A) showed remarkably slow digestion at a 1:5,000 w/w enzyme-to-protein concentration, considering that they are both IDPs. During proteolysis, bands corresponding to apo-gN1 and apo-hN1 remained visible even after 210 min, which given that the limited resolution of the gel greatly obscured any observations. However, during gN1 digestion, an unusual product with lower electrophoretic mobility and an MW of approximately 14 kDa appeared (Fig. S6A). In contrast, the proteolytic pattern of hN1 was different, as there was no product with lower electrophoretic mobility (Fig. S7A). The digestion of holo-gN1 (Fig. S6B) and holo-hN1 (Fig. S7B), although seemingly reduced, was challenging to analyze due to the extended digestion time. A control digestion of apo-hNucb2 (Fig. S7C) at the same enzyme-to-protein ratio (conducted to exclude possible issues with protease activity affecting the resulting digestion pattern) revealed that the apo-state of the hNucb2 protein was digested more rapidly. The first products to appear were approximately 36 and 32 kDa, followed by an increasing accumulation of the products with MWs of approximately 32, 26, 23, and 15 kDa. After 120 min of digestion, the band corresponding to apo-hNucb2 was almost completely digested. In contrast, in the digestion pattern of holo-hNucb2 (Fig. S7D), a strong band corresponding to the holo-state was visible throughout all incubation times. Additionally, the major products were approximately 38 and 36 kDa and were most prominent near the end of proteolysis.

**Fig. 11:**
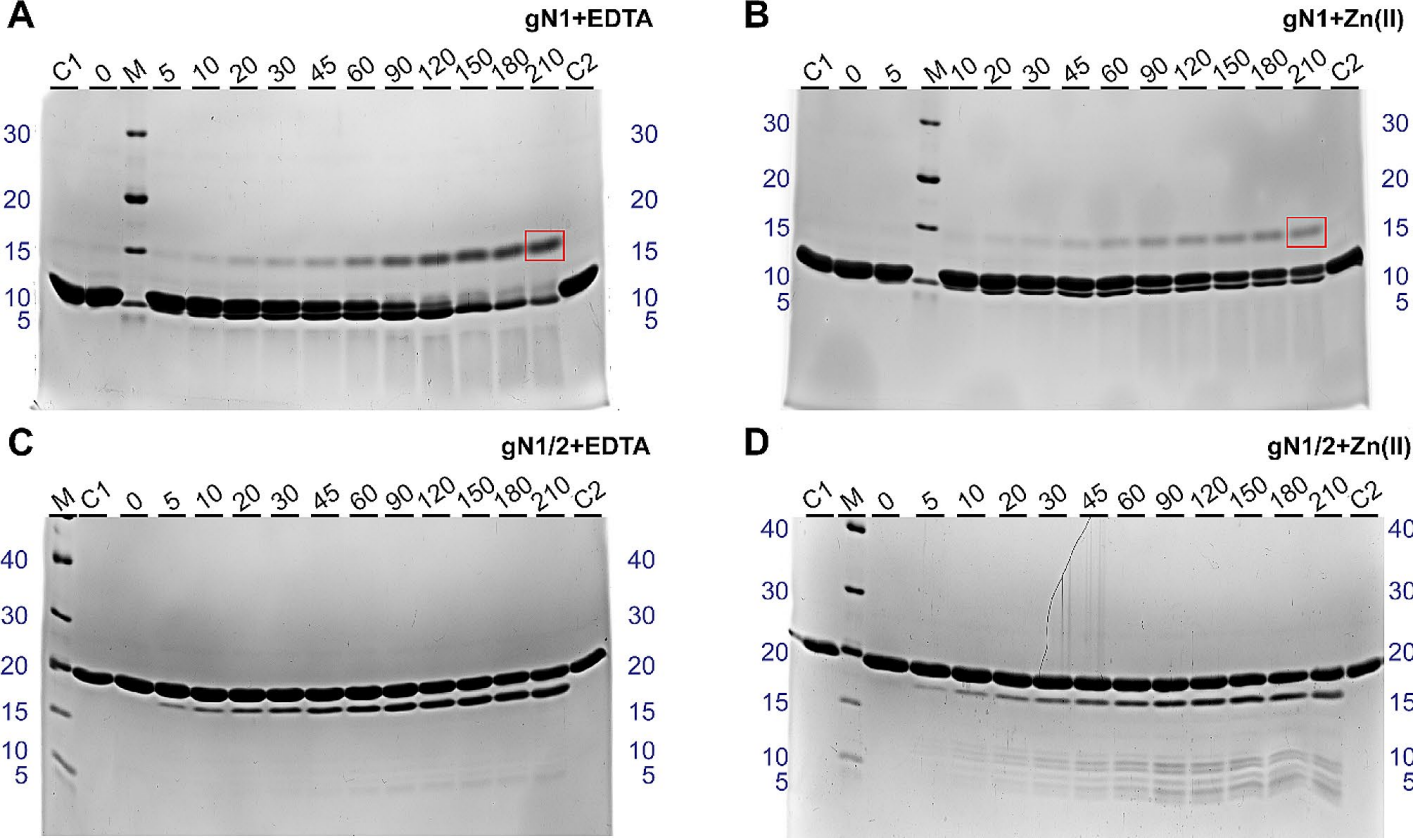
SDS–PAGE analysis of the limited proteolysis of chicken nesfatins. C1, C2 – control samples (without the enzyme) at the beginning and the end of proteolysis, respectively; M – molecular weight marker; 0–210 – samples taken after the digestion time specified in minutes at the top of each panel. gN1 (**A**, **B**) was digested at a 1:1,000 ratio and gN1/2 (**E**, **F**) was digested at a 1:5,000 w/w enzyme-protein ratio. Reactions were performed in the presence of 5 mM EDTA (**A**, **C**) or 500 (**B**)/50 µM Zn(II) (**D**) for gN1 and gN1/2, respectively. The red box indicates the digestion product with a higher apparent MW

**Fig. 12:**
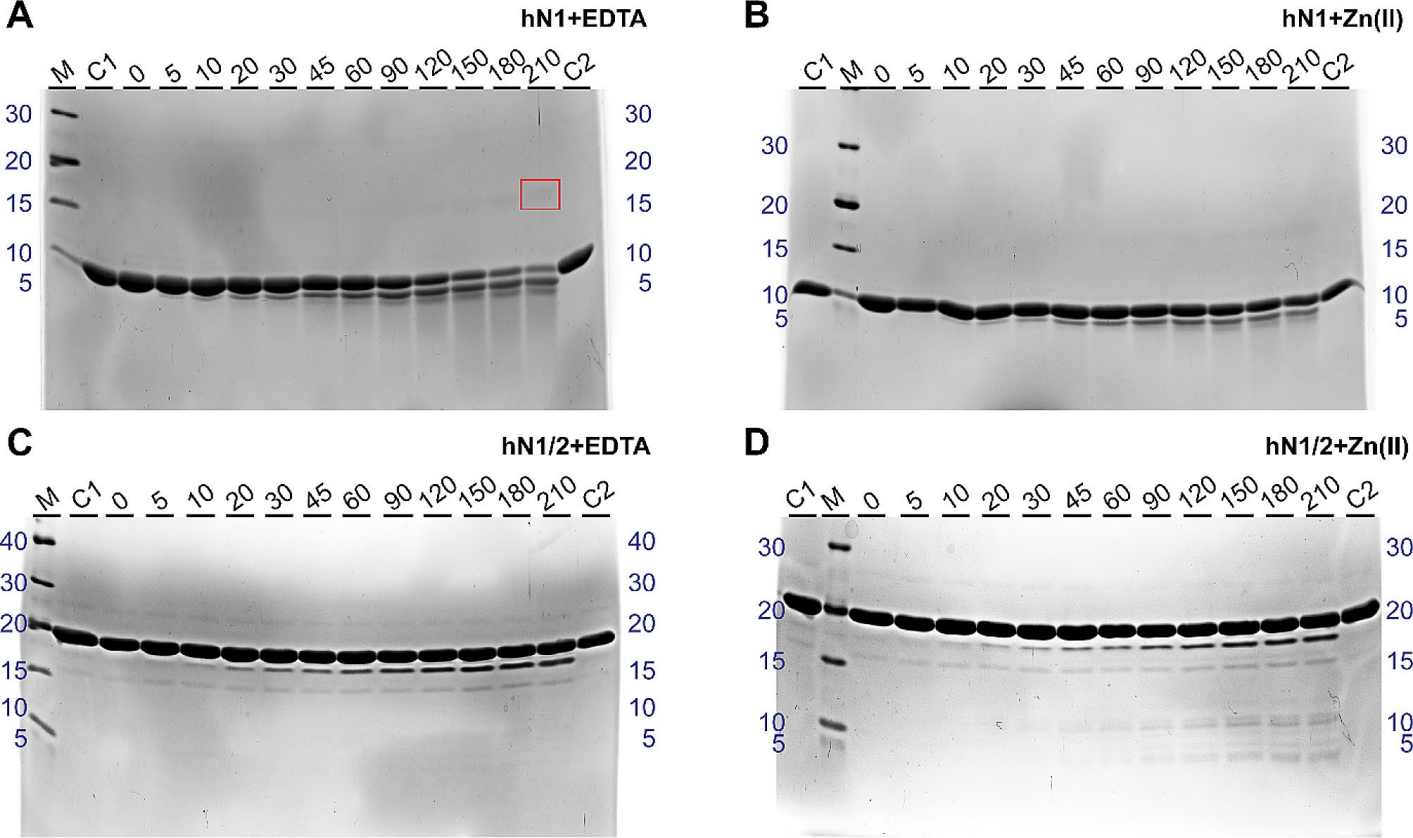
SDS–PAGE analysis of the limited proteolysis of chicken nesfatins. C1, C2 – control samples (without the enzyme) at the beginning and the end of proteolysis, respectively; M – molecular weight marker; 0–210 – samples taken after the digestion time specified in minutes at the top of each panel. gN1 (**A**, **B**) was digested at a 1:1,000 ratio, and gN1/2 (**E**, **F**) was digested at a 1:5,000 w/w enzyme-protein ratio. Reactions were performed in the presence of 5 mM EDTA (**A**, **C**) or 500 (**B**)/50 µM Zn(II) (**D**) for gN1 and gN1/2, respectively. The red box indicates the digestion product with a higher apparent MW

To enhance the visibility of the effect of Zn(II) binding on N1 homologs, as it was obscured by the resolution of the gel and insufficient digestion, additional limited proteolysis experiments of apo- and holo-N1 homologs were performed at a 1:1,000 w/w enzyme-to-protein ratio. Apo-gN1 (Fig. 11A) and apo-hN1 (Fig. 12A) were digested much more efficiently than holo-gN1 (Fig. 11B) and holo-hN1 (Fig. 12B). The main products that accumulated during the digestion of gN1 and hN1 were approximately 7 and 5 kDa, respectively. Moreover, the appearance of the uncommon band of higher MW was even more pronounced during the course of digestion of gN1. This band was still visible, although not as distinct, in holo-hN1. However, during the digestion of apo-hN1, only a very faint but defined band with a MW of approximately 14 kDa appeared in the gel (Fig. 12B). To investigate whether only peptides resulting from the digestion of gN1 were able to form a band of higher MW and to observe the full degradation of gN1, additional experiments were performed. For this purpose, apo- and holo-gN1 were digested in freshly prepared samples for 24 h (Fig. S8A) or after prior maturation of the sample at 20 °C for 24 h, (Fig. S8B). In both cases, full digestion of apo- and holo-gN1 could be achieved (Fig. S8). The mature control and digestion samples seemed to have more faint, barely visible bands of higher MWs, including the 14 kDa band (Fig. S8B). To gain more insight into the content of the 14 kDa band, it was excised from the gel and subjected to MS identification. The identified peptides (data not shown) were proven to be of gN1 origin and covered 86% of its amino acid sequence, with two peptides missing from the C-terminus.

The effect of Zn(II) binding on N1/2 homologs was once more in strong contrast to the effects observed for the respective N1 fragments. Apo-gN1/2 (Fig. 11C) and apo-hN1/2 (Fig. 12C) were less prone to digestion than were holo-gN1/2 (Fig. 11D) and holo-hN1/2 (Fig. 12D), as judged by the amount and intensity of the proteolytic products. Particularly, for both apo-N1/2 homologs, the main product being formed was approximately 18 kDa. There was also a slight increase in the intensity of the band approximately 15 kDa for apo-hN1/2. The proteolytic patterns of holo-gN1/2 and holo-hN1/2 also contained the most prominent band with a MW of approximately 18 kDa. However, the proteolytic pattern of holo-gN1/2 contained more peptides in the 9–12 kDa range (Fig. 11D). Similarly, in the digestion pattern of holo-hN1/2, additional bands at approximately 15, 11, and 5 kDa could be observed (Fig. 12D).

Thus, the effects of Zn(II) interacting with nesfatins revealed by HDX-MS (see Sect. 2.5) were once more emphasized here. Apparently, structurization of the M30 region in gN1 and hN1 led to decreased susceptibility to proteolysis. In contrast, the exposure of the M30 region and two additional regions in gN1/2 and hN1/2 (see Sect. 2.5) led to their increased proteolytic processing. Moreover, the appearance of the band of higher MW in the proteolytic patterns of gN1 and hN1 clearly underlined the context-dependent nature of their aa sequence.

### Do holo-gN1 and holo-hN1 contain an amyloid fold?

**Fig. 13:**
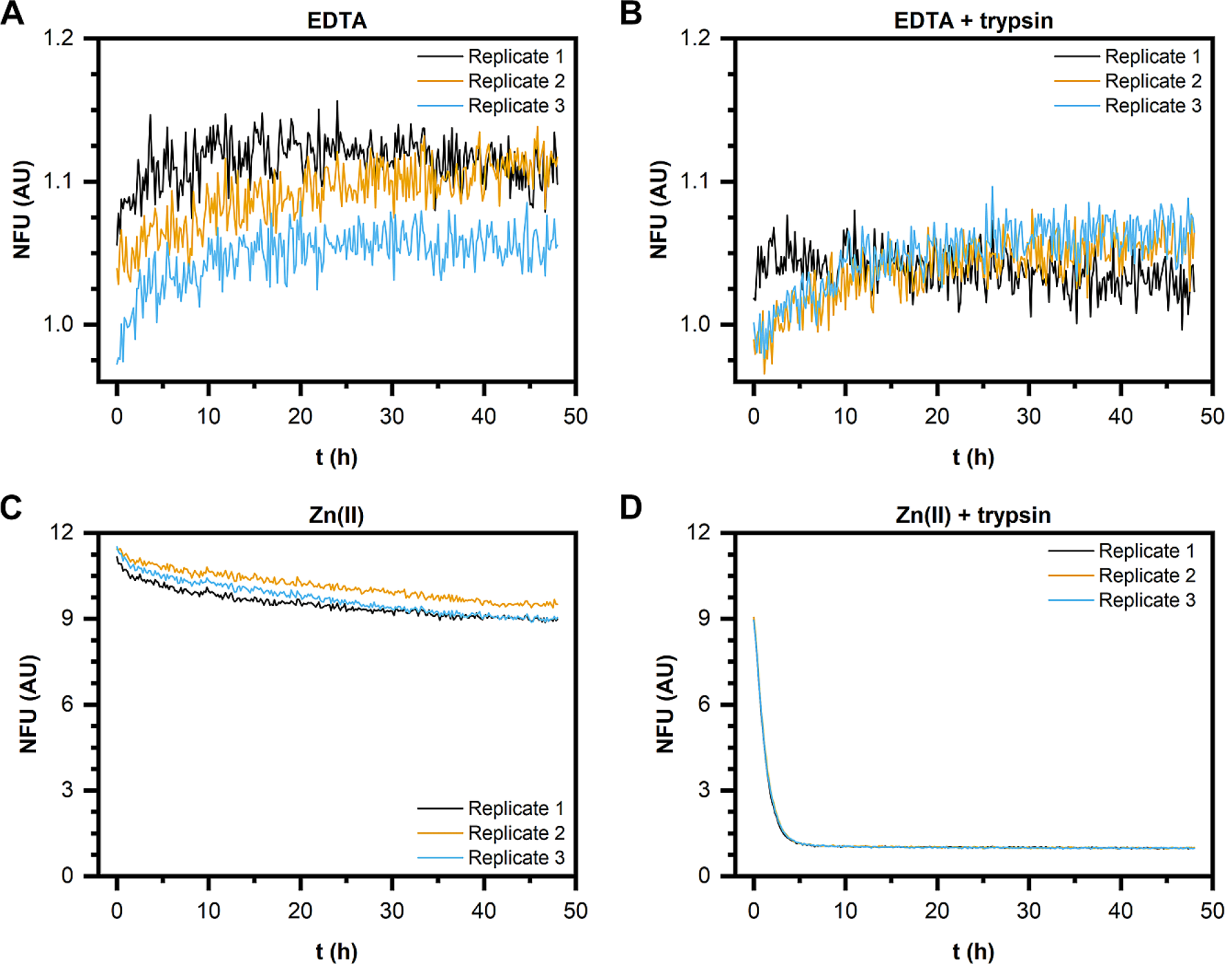
Aggregation kinetics of gN1 monitored by ThT in the presence of 5 mM EDTA (**A**, **B**) and 500 µM Zn(II) (**C**, **D**). Panels (**B**, **D**) show ThT emission in the presence of trypsin

To probe whether the formation of the band of higher MW during the digestion of gN1 (see Sect. 3.4) was associated with gN1 aggregation, ThT binding kinetic and steady-state fluorescence experiments were performed. ThT is a fluorophore that, upon binding to amyloid structures, undergoes a dramatic shift in the fluorescence emission maximum from 450 to 482 nm with a simultaneous increase in its intensity by several fold [49–51]. In the kinetic studies, apo-gN1 (Fig. 13A and B) and holo-gN1 (Fig. 13C and D) were incubated with ThT for 48 h either in the absence (Fig. 13A and C) or presence (Fig. 13B and D) of trypsin. Both apo-gN1, treated and untreated with the protease, showed basal fluorescence compared to that of the buffer (NFU oscillating at approximately 1). In contrast, there was a very high initial NFU value in the absence (Fig. 13C) and presence (Fig. 13D) of trypsin for holo-gN1. However, the NFU in the presence of the protease decreased over time following a hyperbolic trend, contrary to the much more stable ThT fluorescence of the untreated samples.

**Fig. 14:**
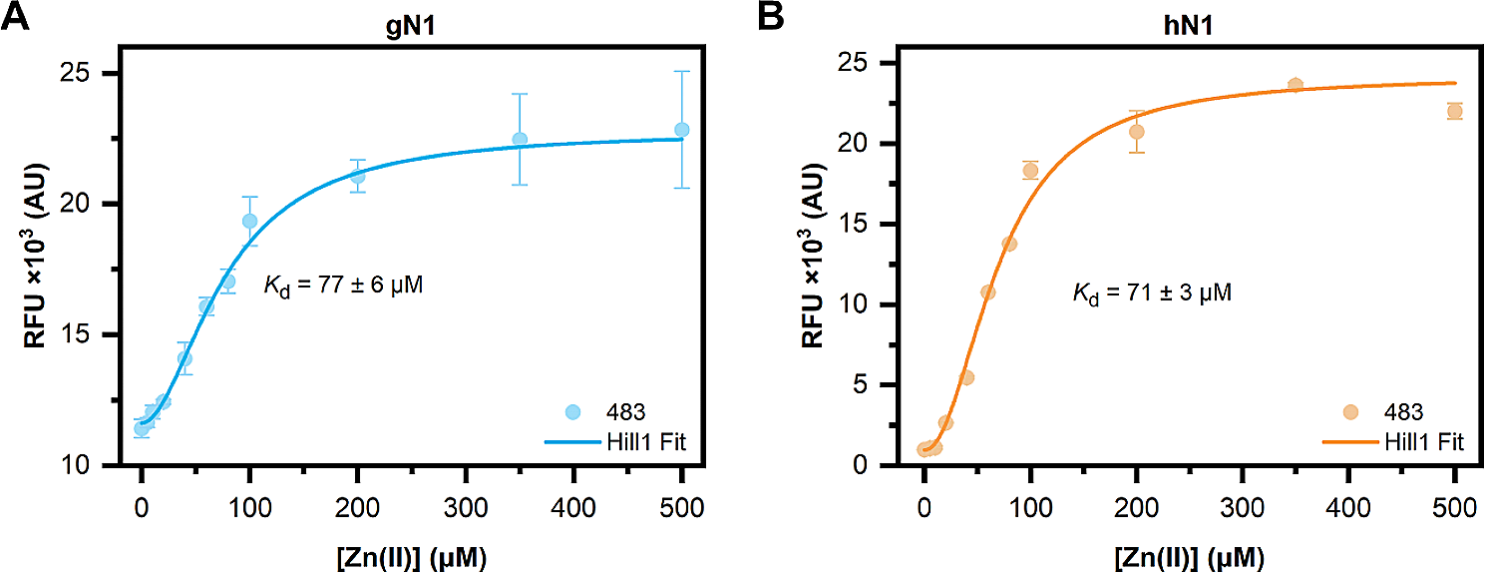
Titration of (**A**) gN1 and (**B**) hN1 (10 µM of each) with Zn(II) in the presence of 5 µM ThT. The data represent the average ± SD from the RFU at 483 nm (circles) and its Hill fit (solid line)

The initial kinetics of ThT binding experiments suggested that the fluorophore was bound by holo-gN1. To examine in more detail whether the binding was indeed Zn(II)-dependent and conserved among homologs, steady-state fluorescence experiments of chicken and human N1 and N1/2 titrated with Zn(II) were performed. The results showed a prominent and Zn(II) concentration-dependent increase in RFU at 483 nm for both gN1 (Fig. S9A) and hN1 (Fig. S9B). The data at this wavelength, fitted against the Hill model, resulted in *K*_d_ values of 77 ± 6 and 71 ± 3 µM for gN1 (Fig. 14A) and hN1 (Fig. 14B), respectively. The data obtained for N1/2 homologs, however, were in sharp contrast to the results obtained for the respective N1 fragments. First, the emission maxima for gN1/2 (Fig. S9C) and hN1/2 (Fig. S9D) were observed at approximately 476 nm and decreases in a Zn(II) concentration-dependent manner.

The binding of ThT, which is an amyloid-specific probe, strongly suggested that holo-N1 homologs might contain an amyloid fold. Since N1s are neuropeptides abundant in the central nervous system, the presence of an amyloid fold suggests their involvement in neurodegenerative processes. However, the nature of their contribution to these processes remains unclear. It is also worth noting that the two-faced effect of Zn(II) on chicken and human N1 and N1/2 could once again be seen.

## Discussion

Given the high degree of homology between nesfatins, it would be reasonable to anticipate similar molecular properties among their homologs. Although initial studies on the structure of human nesfatins [28] have been conducted, our focus in this paper was primarily on gN1. Nevertheless, based on in silico predictions, we expected numerous similarities between chicken and human homologs. Surprisingly, however, we observed significant differences in the molecular properties of chicken homologs across all experiments conducted. These discrepancies suggest that certain functions of Nucb2/nesfatin may be species specific, potentially resulting in variations in their physiological functions among different species.

The CD experiments aimed to analyze the secondary structure content of chicken nesfatins, as described in Sect. 2.2.1. The data obtained confirmed that gN1 belongs to the family of IDPs, which contain approximately 54 ± 2% of IDRs. Notably, the contents of antiparallel *β*-sheets and turns were estimated to be approximately 18 ± 0.4% and 16.0 ± 0.2%, respectively. Despite the presence of residual structures in both chicken and human N1 homologs, they exhibited a predominance of a coil-like structure, as indicated by their positioning on the double-wavelength plot (Fig. S2). Therefore, we demonstrated the conservation of the IDP character of N1 between chicken and human homologs, which likely extends to other N1 and NLP homologs due to their highly conserved amino acid sequence. IDPs, which are unstructured and ubiquitous proteins, play crucial roles in numerous fundamental biological processes and have been extensively studied [52–54]. Thus, the preservation of the IDP character of the multifunctional N1 underscores its biological significance. Moreover, recent research has begun to explore the expression patterns of Nucb2/N1 in other species such as dogs [55–57], pigs [57, 58], and cats [57]. These studies are of special importance because elucidating differences in nesfatin functions among species might be crucial for deciphering the physiological role of N1 and other fragments. Therefore, the IDP character of N1 (in contrast to the mosaic structures of N1/2 and Nucb2 precursors) may be a prerequisite for its pleiotropic mode of action across species. On the other hand, the analysis of the CD data of gN1/2 revealed stark differences compared to gN1. Deconvolution of the gN1/2 spectra indicated that an IDR content of approximately 41 ± 2% and *α*-helix content of approximately 40 ± 2%, were the most abundant. These results were consistent with the IDR predictions (see Sect. 2.1). Additionally, a notable amount of turns was observed in the secondary structure of gN1/2, with a value of 12.0 ± 0.4%. The mosaic character of gN1/2 mirrored that of hN1/2, as discussed previously [28], and was further investigated for both homologs in this study. Furthermore, the disparities between the structures of gN1 and gN1/2 once more underscored the fact that the proteolytical processing of Nucb2 to N1 by PCs, and in turn, unleashing the disorder propensity of the latter, might facilitate its multiple interactions, which are characteristic of IDPs [59] and may partially explain its astonishingly diverse range of functions.

In a subsequent investigation, chicken nesfatins were examined for secondary structure alterations upon treatment with Ca(II) and Zn(II), given the reported binding affinity of both ions to gNucb2 and hNucb2 [27]. Interestingly, we observed no discernible changes in the secondary structure of gN1 and gN1/2 following Ca(II) treatment (Fig. S3), consistent with findings for their human counterparts. This suggests that Ca(II) binding is a characteristic exclusive to Nucb2/N3 [26, 60]. Our investigations revealed conserved Zn(II)-sensing abilities between chicken and human N1 homologs, as detailed in Sect. 2.2.2. Upon Zn(II) treatment, we observed a concentration-dependent increase in the *α*-helical content of gN1 and a simultaneous decrease in the IDR content. Notably, the apparent *K*_d_ range, derived from fitting of the CD data was consistent with values obtained from the ThT binding assay (refer to Sect. 2.7), as well as with values reported for the hN1 homolog, both in this study (Sect. 2.7) and in previous research [28]. These findings suggest the conservation of Zn(II) binding ability in N1 peptides, along with similar binding affinities. However, notable differences emerged between gN1 and hN1 regarding their cooperativity in Zn(II) binding, with gN1 exhibiting less cooperativity based on the Hill parameter value of 1, which produced the best fit. This discrepancy hints at possible differences in the binding mode of Zn(II) between the homologs, despite their highly conserved amino acid sequences. In contrast, Zn(II) had a distinct effect on gN1/2 compared to gN1 during the CD experiments. Initially, there was no discernible change in CD signal intensity under Zn(II) treatment for gN1/2. However, similar to the observations with the hN1/2 homolog [28], a significant decrease in the CD signal intensity was observed in gN1/2 upon surpassing a threshold concentration of 50 µM Zn(II) (refer to Sect. 2.2.2). This process exhibited high cooperativity, emphasizing the dual nature of the proprotein resulting from the different effects of N1 and N2 when covalently bound [28]. Notably, the intensities of the CD signals at 208 and 222 nm were almost equal at Zn(II) concentrations of 75 and 100 µM, a phenomenon not observed for hN1/2 [28]. This observation points to possible differences in the binding mode of Zn(II) by the chicken proprotein (gN1/2), which was further substantiated by subsequent experiments. Hence, initial binding of Zn(II) might induce structural changes and lead to subsequent precipitation and/or aggregation of the peptide in a cooperative manner. Thus, to prevent further precipitation of gN1/2, a Zn(II) concentration of 50 µM corresponding to a CD signal loss above 80% was used (refer to Fig. 4E).

To assess the impact of Zn(II) binding on the thermal stability of nesfatin homologs, temperature-dependent CD experiments were conducted (refer to Sect. 2.2.3). Under Zn(II) treatment, we observed a Zn(II)-dependent decrease in the Tm values of gN1 and hN1. Particularly noteworthy was the strongest Tm difference observed for the hN1 homolog, once again suggesting possible disparities in the interaction with the ion between the homologs. For N1 fragments, changes in the Tm were attributed to gradually formed ordered structures being less stable than the disordered apo-forms, the latter of which, as expected, did not produce a Tm. It is also notable that the low Tm values of holo-N1 homologs might be because only a small fragment appears to be structured in the presence of Zn(II) in both peptides, as indicated by the HDX-MS results (see Sect. 2.5 and the text below). The most pronounced effects of Zn(II) binding on the Tm were observed for N1/2 chicken and human homologs. The decrease in the Tm value was steeper for N1/2 homologs than for N1 homologs. Moreover, the dual nature of the proprotein was again demonstrated, with melt curves exhibiting a biphasic character (refer to Sect. 2.2.2). Hence, Zn(II) binding appeared to both stabilize and destabilize different portions of the peptides. Furthermore, this might imply a loss of cooperativity in domain interactions in the holo-state. These findings seem to be partially explained by the HDX-MS results, which showed increased exposure of the holo-N1/2 backbone (see Sect. 2.5 and the text below). Notably, similar noncooperativity was reported for apo-hNucb1 by Vignesh et al. [61].

SV-AUC (refer to Sect. 2.3) demonstrated that the disorder-to-order transition of gN1, as revealed by CD spectroscopy, was accompanied by a significant decrease in the hydrodynamic volume of the neuropeptide and its dimerization in a Zn(II)-dependent manner. Intriguingly, similar effects of Zn(II) were previously observed for hN1 [28]. Thus, the compaction and dimerization of holo-N1 were shown to be conserved between chicken and human homologs. This feature might be essential for enabling interactions with a different pool of ligands in vivo than apo-N1 and may constitute one of the mechanisms regulating N1 function. This propensity could extend to other N1 and NLP homologs, providing even more fine-tuned regulation of Nucleobindin signaling. However, in the *c*(s) distribution of holo-gN1 there was a third broad peak indicative of peptide aggregation. This was not previously observed for holo-hN1 under the same conditions and once again pointed to differences in Zn(II) binding between the homologs. Furthermore, the *c*(s) distribution of holo-gN1 exhibited a different ratio of the two main peaks compared to the previously observed 1:1 ratio for the hN1 homolog. Additionally, the *c*(s) distribution of gN1/2 appeared to be more sensitive to Zn(II) than the hN1/2 homolog [28]. A slight shift in the s_(20,w)_ parameter of holo-gN1/2 was observed, and a second broad peak indicating oligomerization of gN1/2 under Zn(II) treatment appeared, which was not previously observed for the hN1/2 homolog. Thus, once again, the Janus effects of Zn(II) interacting with nesfatins were demonstrated in this study. Moreover, unlike human nesfatins, only gN1 was shown to compete with ZI for Zn(II) binding. Interestingly, none of the chicken homologs were able to bind ANS (data not shown), as previously shown for human nesfatins [28]. Taken together, despite the high conservation of the aa sequence and Zn(II)-sensing abilities, prominent differences in their binding mechanisms that could be species-specific were observed. Additionally, Zn(II)-treated gNucb2 was previously shown by Bystranowska et al. [27] to be more prone to oligomerization and aggregation than hNucb2. This feature was shown here to be preserved in gN1 and gN1/2.

The HDX-MS experiments revealed the disordered nature of apo-gN1 and apo-hN1 homologs, both showing complete H/D exchange after 10 s. However, under Zn(II) treatment, the M30 region of gN1 and hN1 (Fig. 2) emerged as the most protected region against H/D exchange (see Sect. 2.5). This finding is particularly intriguing as the M30 region is highly conserved among the Nucleobindin family [5] and has been shown to be solely responsible for the anorexigenic mode of action of N1 [6]. The sensitivity of the M30 region to Zn(II) was also demonstrated in N1/2 fragments for both homologs, as well as in previous studies on gNucb2 and hNucb2 [27]. Moreover, both the PONDR and DynaMine algorithms predicted the M30 region to be ordered (see Sect. 2.1). The highest level of protection was observed for the peptides covering the 39–45 amino acid region of both holo-gN1 (RQVIDVL) and holo-hN1 (KQVIDVL). The increased protection of this region, as indicated by the results presented here, seems to be related to the disorder-to-order transition and/or may constitute a dimerization surface, as suggested by the results of SV-AUC experiments. Interestingly, the region containing the putative Zn(II)-binding motif HFREX_n_H (Fig. 2) [62] was much less protected than the aforementioned highly protected region (R/KQVIDVL). Notably, the KQVIDVL sequence was found to be similar to that of an Agouti-related protein (an orexigenic peptide) and unexpectedly constitutes a crucial part of the N1 anorexigenic core [6]. Thus, it is possible that the concealment of the bioactive core of N1 might alter its anorexigenic effects, and, as discussed earlier, provide an interaction interface for a different pool of ligands. Interestingly, similar effects of Zn(II) binding with a disorder-to-order transition and oligomerization were observed for abscisic acid stress ripening proteins (ASRs) by Hamdi et al. [63]. In contrast, the M30 regions of holo-gN1/2 (33–45) and holo-hN1/2 (33–62) were characterized by a moderate to very strong increase in the H/D exchange rate, which is indicative of their exposure to the solvent. Thus, Zn(II) induces Janus effects on the M30 region by concealing it in free holo-N1 homologs while exposing it in holo-N1/2 homologs. Additionally, there were two regions of holo-gN1/2 (60–96, 117–137) and holo-hN1/2 (72–96, 116–137), which were also characterized by a moderate to very strong increases in H/D exchange. The increased solvent exposure of these regions is in particularly good agreement with the data obtained previously for holo-Nucb2 homologs [27]. Moreover, the same regions of apo-gN1/2 appeared to be more protected than apo-hN1/2, resulting in a Zn(II)-driven increase in H/D exchange even after 2.5 h. This observation is once more consistent with the results obtained for Nucb2 homologs [27]. The strong protection of the M30 region of apo-gN1/2 and apo-hN1/2 raises the question of whether they induce the same anorexigenic effect as N1. The highest exposure observed in the regions encompassing amino acids 60–96 of holo-gN1/2 and holo-hN1/2 is of particular importance, as this region contains the cleavage site recognized by PCs (Fig. 2). These observations further support our previous hypothesis that the N2 fragment functions as a structural element regulating the activity and localization of Nucb2 and nesfatins [28]. Moreover, the Golgi apparatus is one of the storage sites for Zn(II), especially in secretory cells such as B cells [64]. Since both Nucb2 and PC1/3 undergo maturation in this compartment, processing of Nucb2/N1/2 in this organelle might be responsible for the trafficking of nesfatins, as discussed earlier [28]. The concentration of free Zn(II) is tightly regulated and maintained in the sub- to nanomolar range [65]. However, the levels of Zn(II) can reach the micromolar range in certain cellular compartments, such as the Golgi apparatus [64] and glutamatergic neurons [66]. This variation in Zn(II) levels could explain the high *K*_d_ values of nesfatin homologs and their potential to exert different functions in a Zn(II)-abundant milieus. In vivo, adopting different conformations and exposing different domains in a Zn(II)-dependent manner seems to be part of the regulatory framework of Nucleobindin activity. This may account for the auto and paracrine effects of Nucb2/N1 and Nucb1/NLP and their involvement in a wide range of physiological functions. The results of limited proteolysis experiments (see Sect. 2.6) further support this hypothesis. Both holo-N1 homologs and holo-hNucb2 were characterized by decreased susceptibility to trypsin proteolysis, indicating the structurization effects of holo-N1 homologs. Surprisingly, despite being members of the IDP family, both apo- and holo-N1 homologs are poor protease substrates and are processed significantly slower than apo-Nucb2s at the same enzyme-to-protein ratio, as shown in this paper and previously [26]. Only after increasing the enzyme-to-protein ratio five times could the protective effect of Zn(II) binding by N1 homologs (resulting from the structurization of the peptides) be fully observed. This finding underscores the unique and distinct properties of each fragment involved in Nucb2 processing, which undoubtedly has functional implications. Moreover, differences in the digestion patterns between holo-N1 homologs indicate distinct conformations of the holo-states. Additionally, there was an unusual proteolytic product of higher apparent MW observed in the digestion pattern of N1 homologs. Based solely on the MW, it was initially speculated that the anomalous product might be an acyl-enzyme intermediate [67]. However, MS identification revealed that the higher molecular weight band was purely of N1 origin. Thus, the increased gel retardation could be attributed to the loss of residual structure in the N1 fragment after cleavage, leading to an increase in its IDP characteristics, particularly affecting its binding affinity with SDS [68]. This hypothesis is strengthened by the context-dependent nature of the nesfatin amino acid sequence, as demonstrated in this paper and previously, where every alteration or removal of amino acid residues from Nucb2 resulted in different molecular properties of the products. Surprisingly, the product of apo-hN1 digestion also exhibited poor dye binding, and the digestion of apo-gN1 appeared to progress faster than that of the human homolog, further highlighting the differences between the N1 homologs. Furthermore, the Zn(II)-driven exposure of the N1/2 backbone observed by HDX-MS was corroborated by limited proteolysis. There was a significant increase in the amount and intensity of bands resulting from the proteolysis of holo-N1/2 compared to the apo-state of the homologs, which were largely protected from hydrolysis. This observation further supports the hypothesis of Zn(II)-induced processing and/or trafficking of the preproprotein at the Golgi apparatus. Notably, Nucb1 was recently identified as a Ca(II)-dependent Golgi sorting protein [69], suggesting that similar effects may be exerted by Nucb2, possibly acting in concert with Zn(II)-induced effects. However, the precise role of Nucb2/nesfatins in Zn(II) sensing and their involvement in the Golgi apparatus require further investigation.

Finally, we studied the aggregation of the products of gN1 digestion in the presence of ThT (see Sect. 2.7). Apo-gN1 exhibited basal ThT fluorescence, indicating poor binding of the fluorophore. However, ThT fluorescence in the presence of holo-gN1 significantly increased in the NFU, and decreased in a hyperbolic manner in the trypsin-containing samples. This suggested that the higher molecular weight product formed during limited proteolysis was not associated with subsequent amyloid structure formation. However, the initial increase in NFU indicated the presence of such a fold change under Zn(II) treatment, which gradually decreased as digestion progressed. This was further supported by steady-state fluorescence experiments for both homologs titrated with Zn(II) (see Sect. 2.7). We observed a strong concentration-dependent increase in RFU under Zn(II) treatment for N1 homologs and a decrease for N1/2 homologs. The data obtained for N1 homologs were fitted, and the calculated *K*_d_ values were consistent with the results obtained in this paper and previously for human homologs. This indicated that the formation of the ThT-binding core is Zn(II)-driven for N1 homologs. In turn, the data obtained for N1/2 homologs did not exhibit a trend that could be fitted. Thus, under specific conditions, Nucb2/N1 may be involved in the pathogenesis of neurodegenerative diseases since, as both are localized in the brain. Further studies are required to determine whether Nucb2/N1 exhibit chaperone-like effects by adopting an amyloid fold conformation that allows binding to and inhibition of fibril growth, similar to Nucb1 [18].

The findings presented here offer valuable insight into the molecular properties of highly conserved chicken and human N1 and N1/2 homologs, which are products of Nucb2 processing. We concluded that both chicken and human N1 homologs belong to the coil-like IDP family, while N1/2 homologs exhibit a mosaic structure. The IDP nature and Zn(II) sensing abilities of nesfatins appear to be well-conserved across species. However, the apo- and Zn(II)-bound forms of the N1 and N1/2 homologs seem to adopt different conformations and exhibit distinct modes of Zn(II) sensing. Thus, despite the high homology of Nucb2/nesfatins, some of their characteristics appear to be species-specific. Additionally, the M30 region and the region recognized by PCs were shown to be sensitive to Zn(II) in Nucb2/nesfatin homologs, suggesting a significant regulatory role for this ion. Moreover, both chicken and human N1 homologs were found to contain an amyloid fold, suggesting their potential involvement in neurodegenerative processes. Therefore, further research on the unique properties of Nucb2/nesfatins in different species, as well as their functional relationships, is essential.

### Electronic supplementary material

Below is the link to the electronic supplementary material.

Supplementary Material 1

## Data Availability

The datasets generated during and/or analyzed during the current study are available from the corresponding author upon reasonable request.

## Abbreviations

aa: Amino acid residue(s)
CD: Circular dichroism
FFC: Fluorescence fold change
HDX-MS: Hydrogen-deuterium exchange coupled with mass spectrometry
IDPs: Intrinsically disordered proteins
IDRs: Intrinsically disordered regions
IMAC: Ion metal affinity chromatography
M30: Middle fragment of nesfatin-1
MRE: Mean residue ellipticity
N1: Nesfatin-1
N1/2: Nesfatin-1 and nesfatin-2 conjoined in a head-to-tail manner
NFU: Normalized fluorescence unit
NLP: Nesfatin-1-like peptide
Nucb1: Nucleobindin-1
Nucb2: Nucleobindin-2
PC1/3: Prohormone convertase 1/3
PC2: Prohormone convertase 2
RFU: Raw fluorescence units
SV-AUC: Sedimentation-velocity analytical ultracentrifugation
ThT: Thioflavin T
ZI: Zincon
