# Supplementary material for: Deciphering the dual nature of nesfatin-1: a tale of zinc ion’s Janus-faced influence
Source: Cell Commun Signal. 2024 May 29;22:298. doi: 10.1186/s12964-024-01675-x (PMC11134965; doi:10.1186/s12964-024-01675-x)
Supplement: Supplementary file 1 — Supplementary Material 1 [file 12964_2024_1675_MOESM1_ESM.docx]

*Supplementary data*

**Deciphering the dual nature of nesfatin-1: a tale of zinc ion's Janus-faced influence**

**Rafał Lenda^1^, Lilia Zhukova^2^, Andrzej Ożyhar^1^, Dominika Bystranowska^1, ✉^**

^1^ Department of Biochemistry, Molecular Biology and Biotechnology, Faculty of Chemistry, Wrocław University of Science and Technology, Wybrzeże Wyspiańskiego 27, 50-370 Wrocław, Poland

^2^ Institute of Biochemistry and Biophysics, Polish Academy of Sciences, Pawińskiego 5a, 02-106 Warsaw, Poland

✉ To whom correspondence should be addressed. E-mail: [dominika.bystranowska@pwr.edu.pl](mailto:dominika.bystranowska@pwr.edu.pl)

**Keywords**

Gallus gallus, chicken, metal cation binding protein, nesfatins, metalloprotein, neuropeptide, satiety molecule, hormone, intrinsically disordered protein

**Figures**


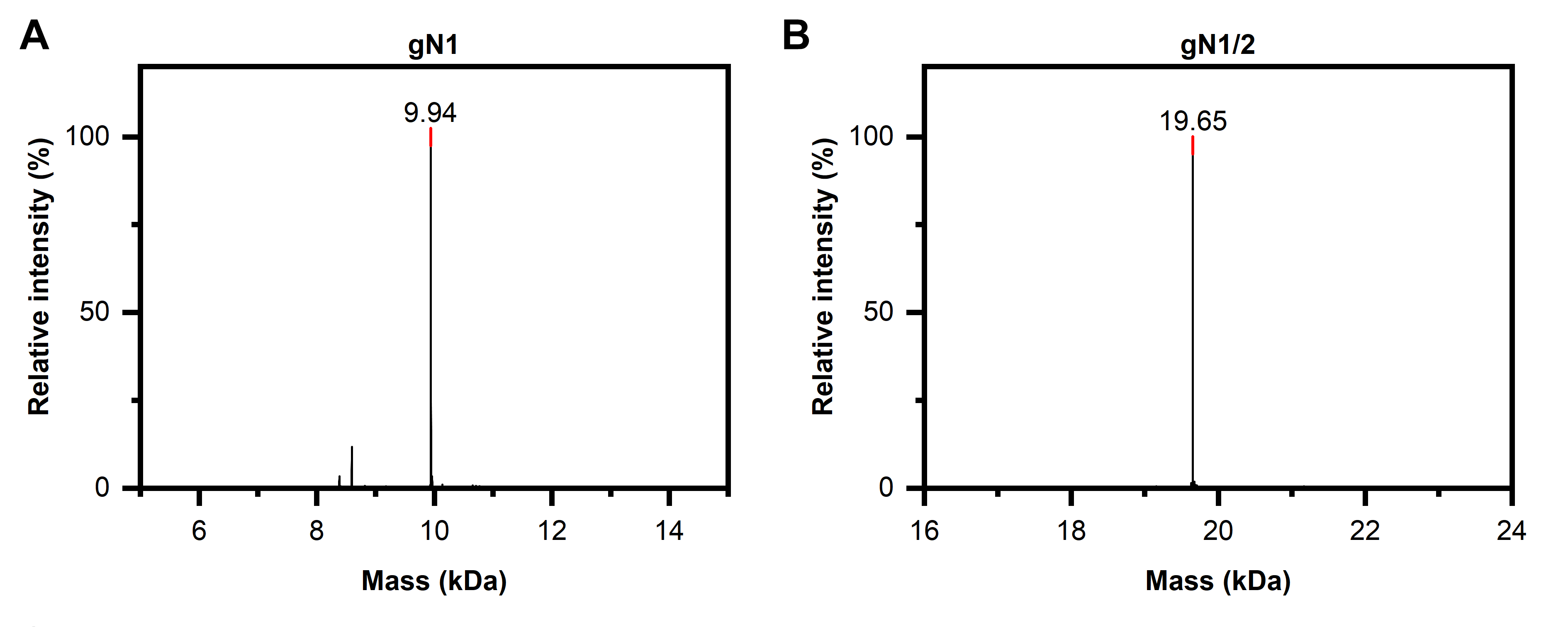


1. MS spectra of (**A**) gN1 and (**B**) gN1/2.


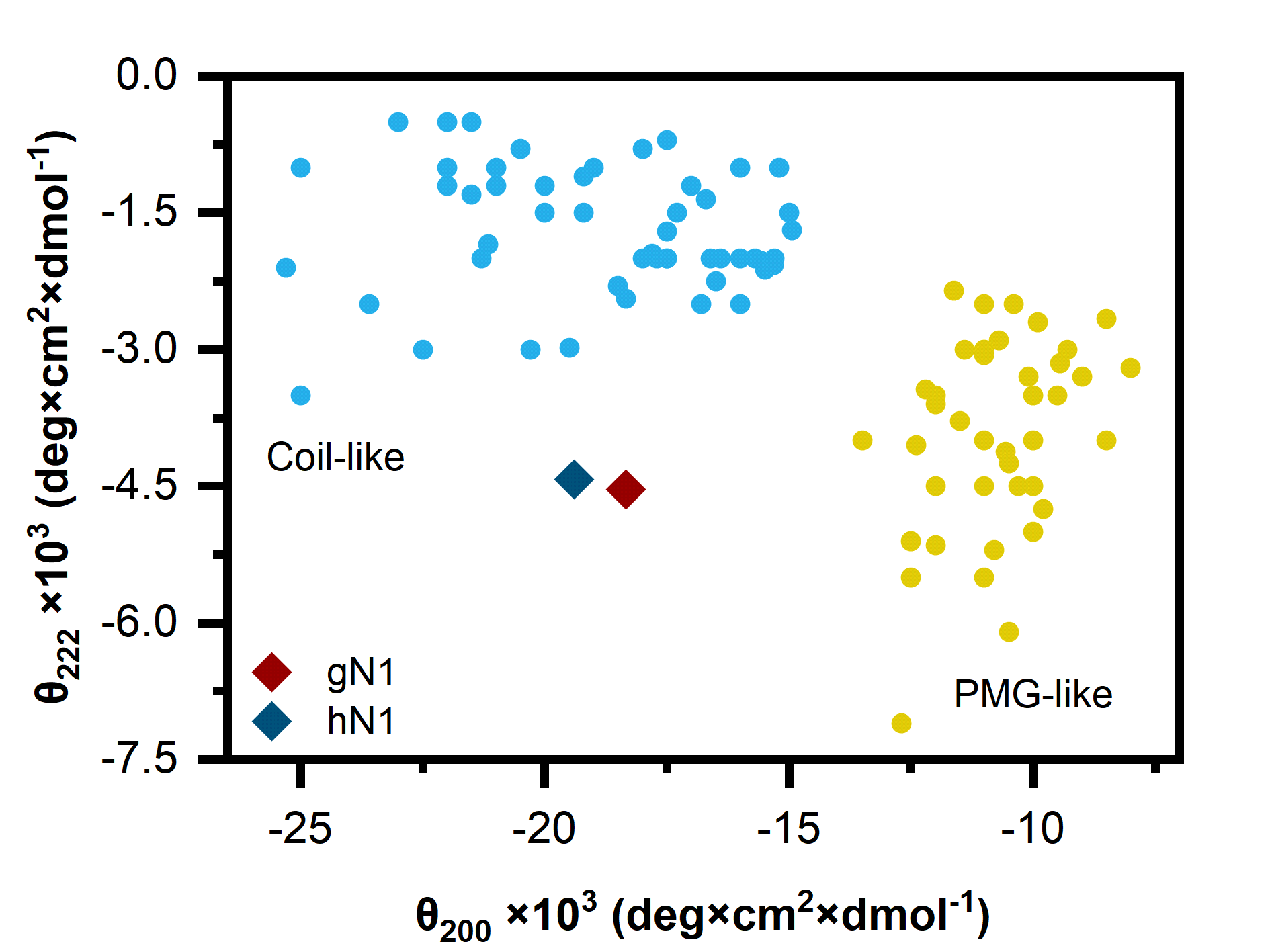


1. The double-wavelength plot of mean residue ellipticity (MRE) at 200 and 222 nm of N1 homologs (diamonds) compared to a set of characterized coil-like and premolten globule (PMG-like) intrinsically disordered proteins (IDPs) (from [66]).


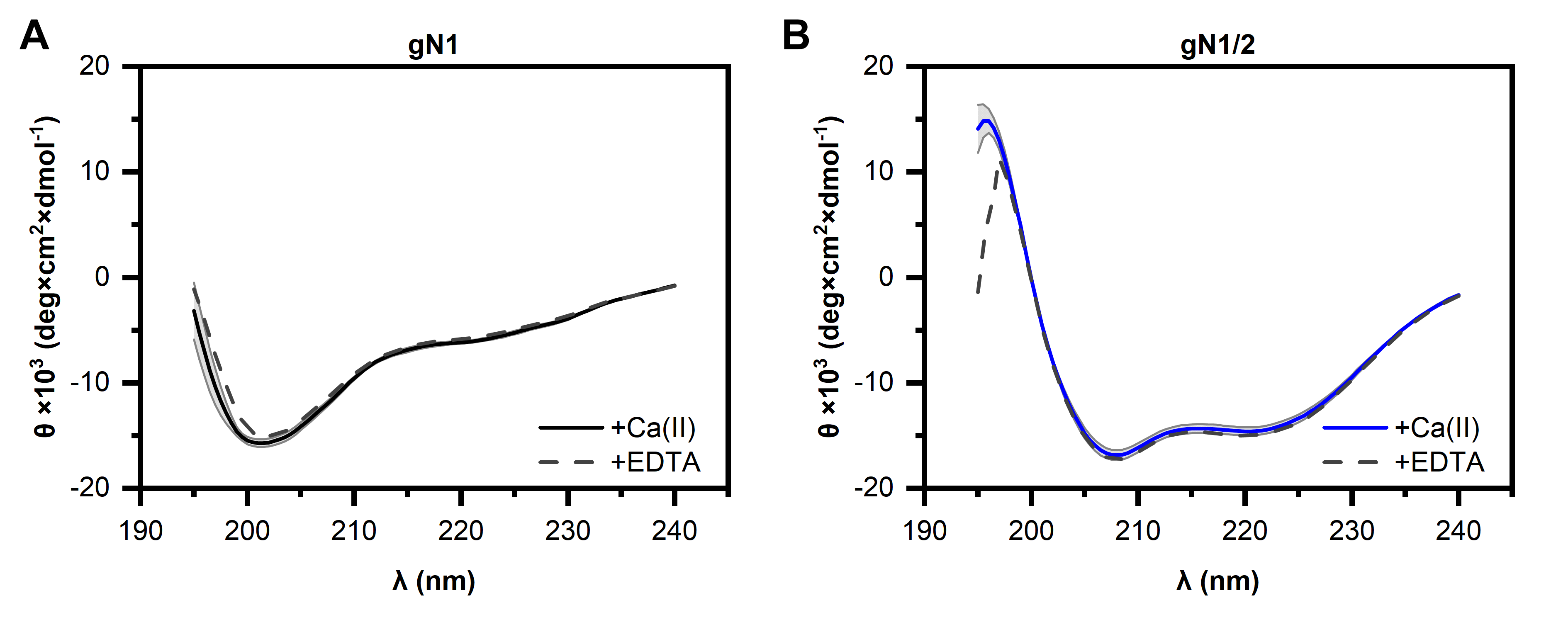


1. Average circular dichroism (CD) spectra of (**A**) gN1 and (**B**) gN1/2 (0.15 mg/ml each) in the presence of 10 mM Ca(II) and 5 mM EDTA depicted as solid and dashed lines, respectively. The gray area represents standard deviation of the +Ca(II) samples.


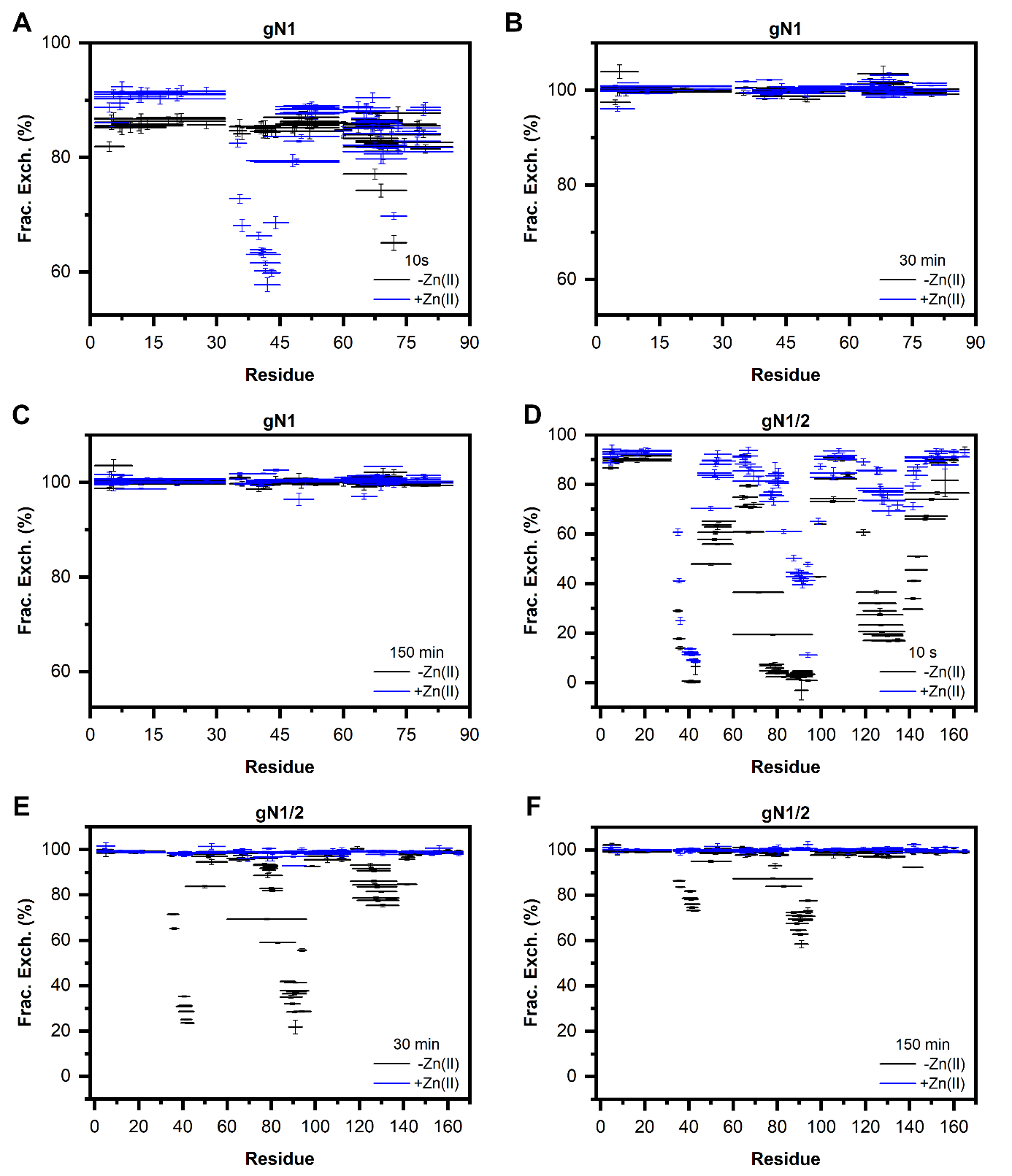


1. Hydrogen-Deuterium Exchange Mass Spectrometry (HDX-MS) analysis of chicken nesfatin-1 (**A**-**C**) and nesfatin-1/2 (**D**-**F**) in the absence/presence of Zn(II) ions at the exposure time specified in each panel.


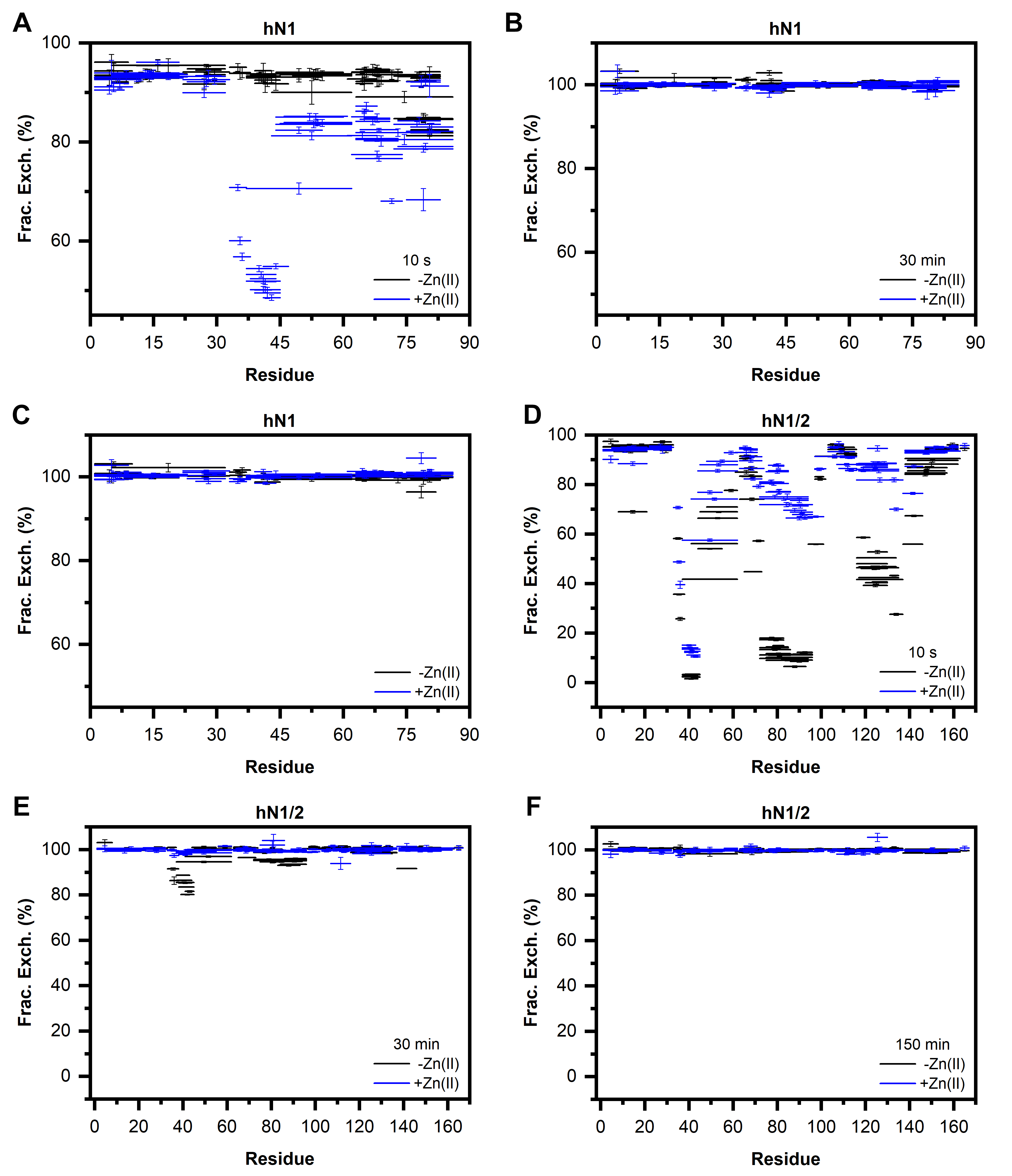


1. HDX-MS analysis of human nesfatin-1 (**A**-**C**) and nesfatin-1/2 (**D**-**F**) in the absence/presence of Zn(II) ions at the exposure time specified in each panel.


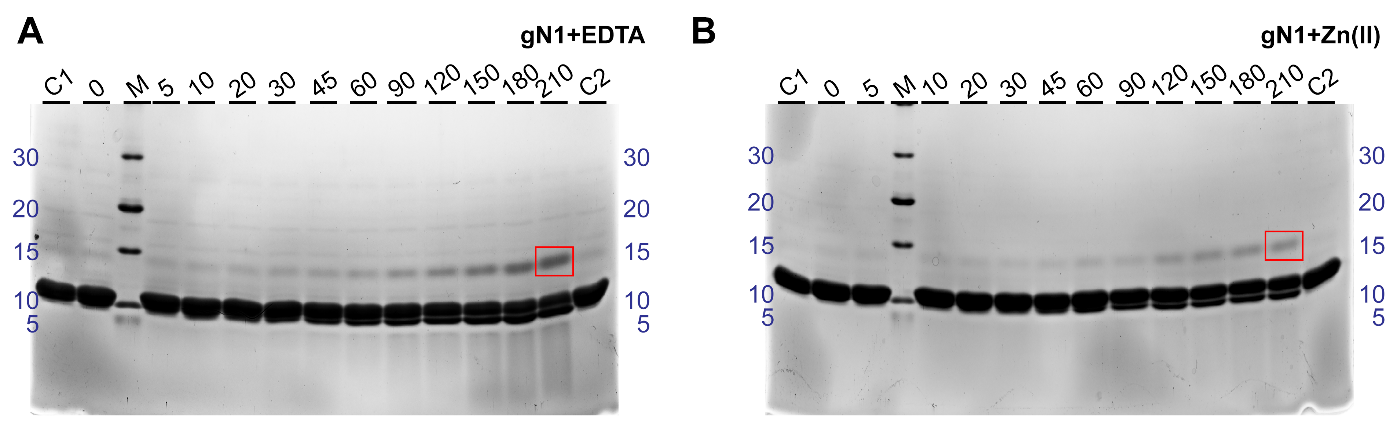


1. SDS–PAGE analysis of the limited proteolysis of gN1. C1, C2 – control samples (without the enzyme) at the beginning and the end of proteolysis, respectively; M – molecular weight marker; 0–210 – samples taken after the digestion time specified in minutes at the top of each panel. gN1 was digested with trypsin at a 1:5,000 w/w enzyme-to-protein ratio in the presence of 5 mM EDTA (**A**) or 500 μM Zn(II) (**B**). The red box indicates a digestion product with an apparently higher MW.


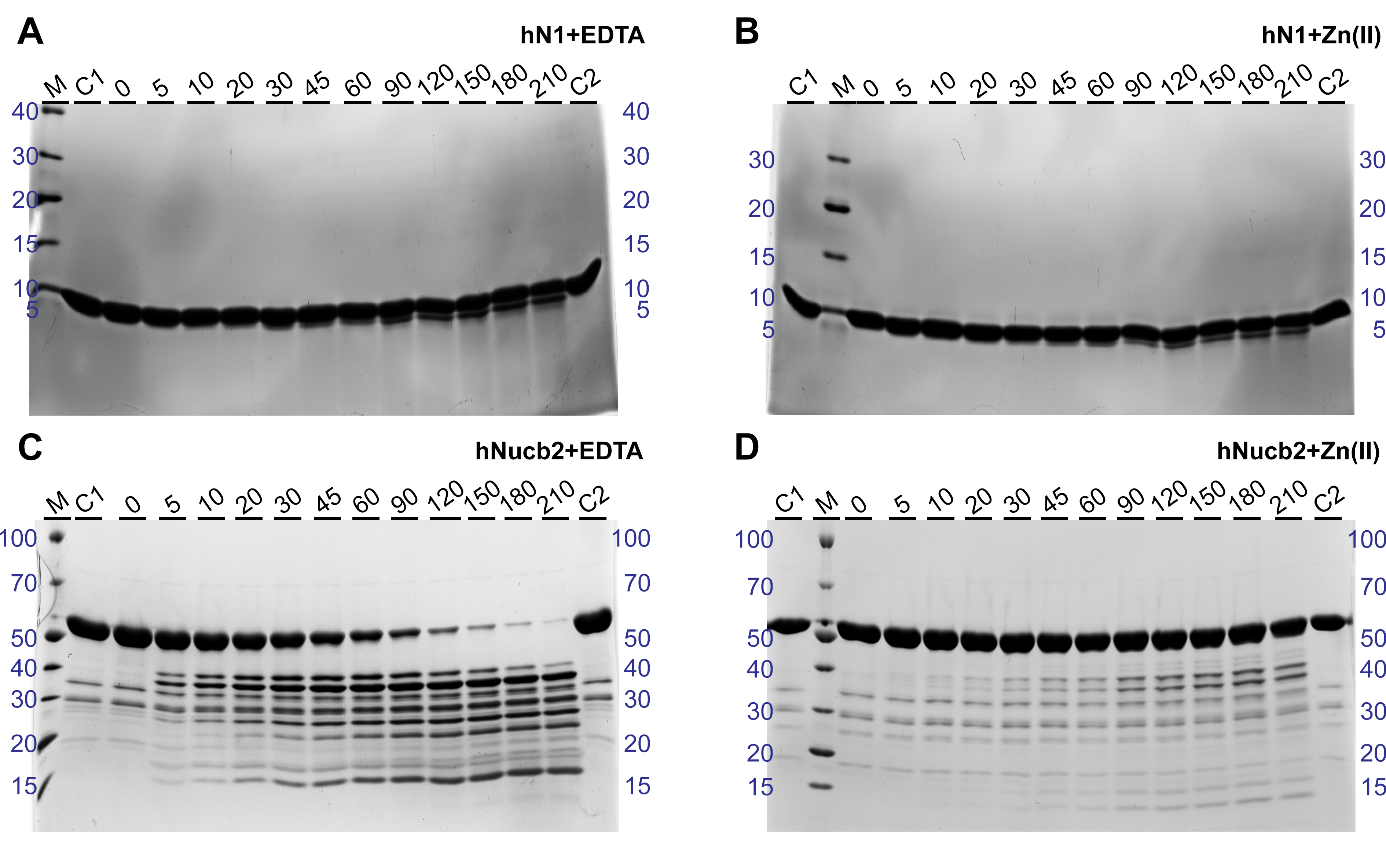


1. SDS–PAGE analysis of the limited proteolysis of human N1 and Nucleobindin-2. C1, C2 – control samples (without the enzyme) at the beginning and the end of proteolysis, respectively; M – molecular weight marker; 0–210 – samples taken after the digestion time specified in minutes at the top of each panel. hN1 and the full-length protein were digested with trypsin at a 1:5,000 w/w enzyme-to-protein ratio in the presence of 5 mM EDTA (**A**, **C**), 500 μM Zn(II) (**B**) or 300 μM Zn(II) (**D**).


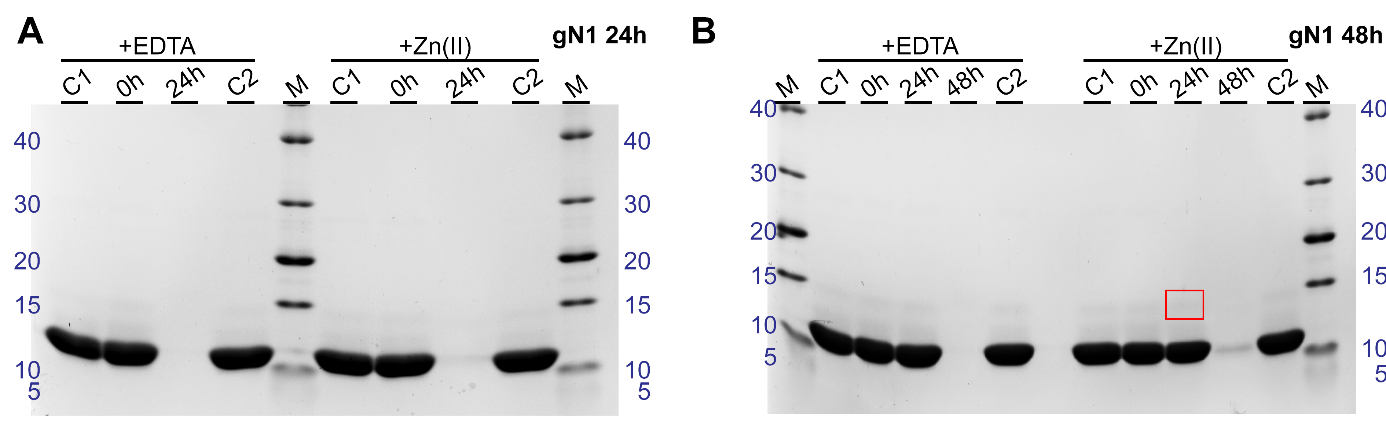


1. Digestion of gN1 by trypsin (at the 1:1,000 w/w enzyme-to-protein ratio) for 24 h. Samples were incubated for 0 h (**A**) and 24 h (**B**) before proteolysis in the presence of EDTA and 500 μM Zn(II). C1, C2 – control samples (without an enzyme) at the beginning and end of the incubation; M – molecular weight marker; 0–48 h – samples taken after the incubation time specified at the top of the lane. The red box indicates a digestion product of apparently higher MW.


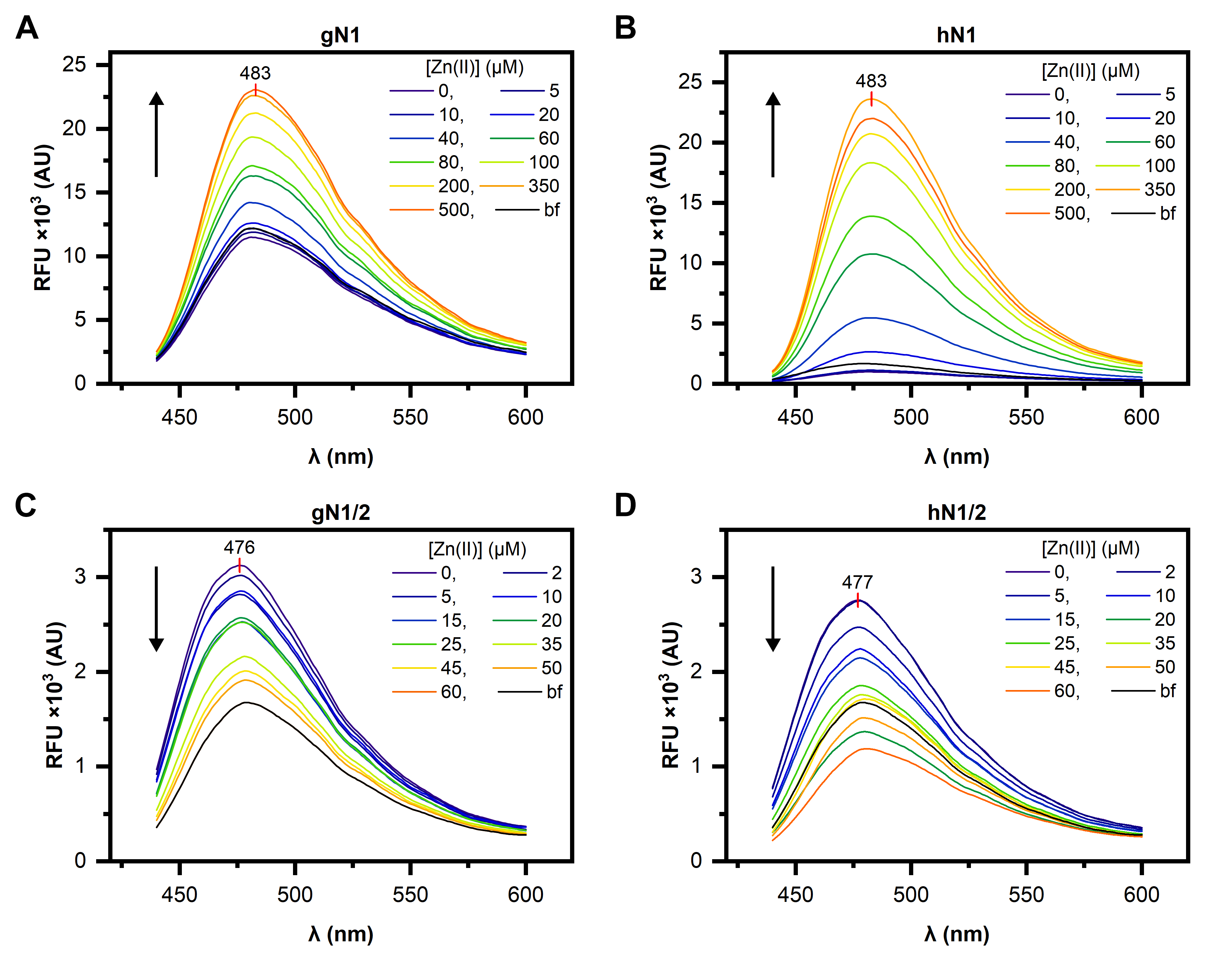


1. Fluorescence emission spectra of ThT (5 μM) in the presence of (**A**) gN1, (**B**) hN1, (**C**) gN1/2, (**D**) hN1/2 (10 μM each) titrated with Zn(II) in the concentration shown in each panel. The RFU of ThT in buffer was marked as bf.

**Tables**

1. Hydrodynamic properties of gN1.

| Protein | c [mg/ml] | Compound | rmsd ×10^3^ | s_(20, w)_ [S] | f/f_0_ | R_h_ [nm] | M_app_ [kDa] (%) |
| --- | --- | --- | --- | --- | --- | --- | --- |
| gN1 | 0.7 | (50 μM) Zn(II) | 8.13 | 1.08  2.16 | 1.61 | 2.38  3.37 | 11.1 (83)  31.4 (17) |
|  | 1.0 |  | 8.76 | 1.05  2.18 | 1.68 | 2.51  3.62 | 11.4 (83)  33.9 (17) |
|  | 1.3 |  | 9.24 | 1.04  2.14 | 1.68 | 2.49  3.58 | 11.2 (83)  33.1 (17) |

The numbers in the round bracket represent the percentage of each fraction relative to the main sedimenting species (100%).
